# PENDISC: A Simple Method for Constructing a Mathematical Model from Time-Series Data of Metabolite Concentrations

**DOI:** 10.1007/s11538-014-9960-8

**Published:** 2014-05-07

**Authors:** Kansuporn Sriyudthsak, Michio Iwata, Masami Yokota Hirai, Fumihide Shiraishi

**Affiliations:** 1 RIKEN Plant Science Center, Yokohama, Kanagawa 230-0045 Japan; 2 Metabolic Systems Research Team, RIKEN Center for Sustainable Resource Science, 1-7-22 Suehiro-cho, Tsurumi-ku, Yokohama, Kanagawa 230-0045 Japan; 3 JST, CREST, Kawaguchi, Saitama 332-0012 Japan; 4 Graduate School of Bioresource and Bioenvironmental Sciences, Kyushu University, Fukuoka , 812-8581 Japan

**Keywords:** Biochemical Systems Theory, Parameter estimation, Mathematical modeling, Metabolomics, Non-linear least squared regression

## Abstract

The availability of large-scale datasets has led to more effort being made to understand characteristics of metabolic reaction networks. However, because the large-scale data are semi-quantitative, and may contain biological variations and/or analytical errors, it remains a challenge to construct a mathematical model with precise parameters using only these data. The present work proposes a simple method, referred to as PENDISC (arameter stimation in a on-mensionalized -system with onstraints), to assist the complex process of parameter estimation in the construction of a mathematical model for a given metabolic reaction system. The PENDISC method was evaluated using two simple mathematical models: a linear metabolic pathway model with inhibition and a branched metabolic pathway model with inhibition and activation. The results indicate that a smaller number of data points and rate constant parameters enhances the agreement between calculated values and time-series data of metabolite concentrations, and leads to faster convergence when the same initial estimates are used for the fitting. This method is also shown to be applicable to noisy time-series data and to unmeasurable metabolite concentrations in a network, and to have a potential to handle metabolome data of a relatively large-scale metabolic reaction system. Furthermore, it was applied to aspartate-derived amino acid biosynthesis in *Arabidopsis thaliana* plant. The result provides confirmation that the mathematical model constructed satisfactorily agrees with the time-series datasets of seven metabolite concentrations.

**Electronic supplementary material:**

The online version of this article (doi:10.1007/s11538-014-9960-8) contains supplementary material, which is available to authorized users.

## Introduction

Comprehensive methods using high-throughput analytical instruments have made it possible to simultaneously measure cellular metabolite concentrations (or their relative quantities referenced by peak intensities or heights) (Fiehn 2002; Sawada et al. 2009; Weckwerth 2003). Using these measured values to construct a mathematical model would enable us to carry out the in silico simulation of metabolic behaviors in various conditions. This would, in turn, allow us to efficiently characterize a metabolic reaction network, resulting in greater potential to further design a desired network. However, not all metabolites in a focused pathway can be simultaneously measured, and the measured metabolite concentrations are not yet sufficiently accurate for the construction of a precise mathematical model, which is due to the following several factors. First, most comprehensive analytical methods for large-scale analysis usually provide only relative quantities, although it is better to use absolute metabolite concentrations to construct a detailed model. Second, in some cases, biological variations and analytical errors are significant. This makes it difficult to identify exact metabolite quantities and show clear tendency of metabolite concentrations changing over a period in which parameter values are estimated in the process of model construction. Third, some important metabolites (e.g., those affecting a metabolic pathway of interest) may be undetectable by simultaneous analytical methods used in metabolomics, so that the mathematical model constructed on the basis of available experimental data may lack essential information.

The simplification of mathematical modeling using power-law representations, such as saturable and synergistic (S)-system or generalized mass action (GMA)-system representations in the framework of biochemical systems theory (BST) (Savageau 1969a, b, 1970; Shiraishi and Savageau 1992; Voit 2013), probably has the potential to overcome the above problems. This is because such modeling techniques allow us to straightforwardly formulate the mathematical equations that describe the time-transient behaviors of metabolite concentrations in a metabolic reaction network by means of only a metabolic pathway map comprised enzymatic reactions and regulatory relationships. In addition, among the widely used kinetic representations, the S-system presents a non-linear representation with the fewest number of kinetic parameters, i.e., rate constants and kinetic orders, which significantly reduces the complexity of constructing and analyzing the model. Even though the S-system equations allow us to set up equations easily with the minimum number of parameters, however, the values of these parameters must be appropriately determined to express metabolic behaviors. For the large-scale metabolic reaction systems, moreover, it is necessary to determine the values of many rate constants and kinetic orders. Thus, parameter estimation is remained as a bottleneck, or limiting procedure, in the process of model construction.

Several methods that use time-series data of metabolite concentrations to estimate the kinetic parameters of the S-system equations have been proposed (Chou 2006; Jia et al. 2011; Kutalik et al. 2007). For example, the decoupling method (Chou 2006) can reduce computational complexity in the parameter estimation. However, since this method uses the slopes of the changes in metabolite concentrations as a part of the relevant objective function, the estimation may strongly depend on the performance of a data-fitting method such as an automated smoother (Vilela et al. 2007), neural network, or polynomial fitting (Voit and Almeida 2004). A combination of the decoupling method with ordinary differential equation decomposition methods has recently been proposed (Jia et al. 2011). This method seems more flexible and easy to predict metabolic behaviors from noisy and incomplete datasets. However, a large number of data may be required to grasp a trend in the dynamic behaviors of metabolite concentrations from their time-series data. Thus, the parameter estimation methods available have advantages and disadvantages and are still under development.

The present study, therefore, proposes a simple method for constructing a mathematical model in an S-system equation model, in which the number of parameters to be estimated is decreased significantly in a special case, and the probable behaviors of unmeasurable metabolite concentrations can also be predicted. A mathematical model is constructed in non-dimensionalized S-system equation form, and the number of unknown rate constants for influxes and effluxes is reduced using the constraints derived from a network structure. Kinetic orders are fixed at a value of 0.5 or $-0.5$, and the remaining rate constants are estimated by fitting calculated values to measured metabolite concentrations.

## Methods

### S-System Equations

The evolution of intracellular metabolite concentrations, $X_{i}$, in a metabolic reaction system can be expressed by

1 $$\begin{aligned} \frac{dX_i}{dt} = \sum _{k=1}^p {v_k} -\sum _{k=1}^q {v_{-k}} \quad (i = 1, 2, \ldots , n), \end{aligned}$$

where $t$ is the time; $v_{k}$ and $v_{-k}$ are the influx and efflux, respectively, of pool $X_{i}$; and $p$ and $q$ are the maximum number of influxes and effluxes. In BST, Eq. (1) is transformed to the following S-system equations:

2 $$\begin{aligned} \frac{dX_i}{dt}=\alpha _i \prod _{j=1}^n {X_j^{g_{ij}}} -\beta _i \prod _{j=1}^n {X_j^{h_{ij}}} = V_i -V_{-i} \quad (i = 1, 2, \ldots , n), \end{aligned}$$

where $V_{i}$ and $V_{-i}$ are the net influx and efflux; $\alpha _{i}$ and $\beta _{i}$ are the rate constants contained in the net influx and efflux, respectively; $g_{ij}$ and $h_{ij}$ are their kinetic orders; and $n$ is the number of dependent variables. The S-system equations consist of two power-law terms, in which the local influxes and effluxes are individually aggregated into a single power-law form (Savageau 1969a, b). Once a metabolic pathway map is given, the symbol $X_{i}$ is assigned to each metabolite concentration, and differential mass balances are taken with respect to each metabolite concentration. This gives the S-system differential equation model expressed by Eq. (2).

### Fundamental Equations for Analysis

When the system has a steady state, Eq. (2) can be transformed to its dimensionless form.

3 $$\begin{aligned} \frac{dx_i}{dt}=A_i \prod _{j=1}^n {x_j^{g_{ij} } } -B_i \prod _{j=1}^n {x_j^{h_{ij} } } \quad (i = 1, 2, \ldots , n), \end{aligned}$$

where $x_{i}$ ($i = 1, 2,\ldots ,n$) are the dimensionless metabolite concentrations with respect to the steady-state metabolite concentrations $X_{i}^{*}$ ($i = 1, 2,\ldots ,n$). These are defined as

4 $$\begin{aligned} x_i =X_i /X_i ^{*}\quad (i = 1, 2, \ldots , n), \end{aligned}$$

and $A_{i}$ and $B_{i}$ are the following dimensionless rate constants for the influx and efflux, respectively,

5 $$\begin{aligned} A_i&= \frac{\alpha _i}{X_i^*}\prod _{j=1}^n {X_j^{*g_{ij}}} \quad (i = 1,2, \ldots , n)\end{aligned}$$

6 $$\begin{aligned} B_i&= \frac{\beta _i}{X_i^*}\prod _{j=1}^n {X_j^{*h_{ij}}}\quad (i = 1, 2, \ldots , n). \end{aligned}$$

Since $A_{i}=B_{i}$ ($i = 1, 2,\ldots ,n$) in a steady-state, Eq. (3) can be further simplified to

7 $$\begin{aligned} \frac{dx_i}{dt}=A_i \left( {\prod _{j=1}^n {x_j^{g_{ij}}} - \prod _{j=1}^n {x_j^{h_{ij}}}}\right) \quad (i=1, 2,\ldots ,n). \end{aligned}$$

A metabolic reaction network may contain linear, branching, and confluent structures and provides the following constraints accordingly.

#### Linear Structure

Consider a metabolic reaction network with a linear structure consisting of the metabolites $X_{i}$ ($i= 1,\ldots ,n$), as shown in Fig. S1 (Supplementary Information 3). The S-system equations for this structure are as follows:

8 $$\begin{aligned} \begin{aligned} \frac{dX_1}{dt}&=\alpha _1 -\beta _1 X_1^{h_{11} } \\ \frac{dX_2 }{dt}&=\beta _1 X_1^{h_{11} } -\beta _2 X_2^{h_{22} } \\&\quad \vdots \quad \quad \quad \quad \;\;\vdots \\ \frac{dX_i }{dt}&=\beta _{i-1} X_{i-1}^{h_{i-1,i-1} } -\beta _i X_i^{h_{ii} } \\&\quad \vdots \quad \quad \quad \quad \;\;\vdots \\ \frac{dX_n }{dt}&=\beta _{n-1} X_{n-1}^{h_{n-1,n-1} } -\beta _n X_n^{h_{nn} } \\ \end{aligned} \end{aligned}$$

The dimensionless form of Eq. (8) is expressed as

9 $$\begin{aligned} \begin{aligned} \frac{dx_1 }{dt}&=A_1 (1-x_1^{h_{11} } ) \\ \frac{dx_2 }{dt}&=A_2 (x_1^{h_{11} } -x_2^{h_{22} } ) \\&\quad \vdots \quad \quad \quad \quad \;\;\vdots \\ \frac{dx_i }{dt}&=A_i (x_{i-1}^{h_{i-1,i-1} } -x_i^{h_{ii} } ) \\&\quad \vdots \quad \quad \quad \quad \;\;\vdots \\ \frac{dx_n }{dt}&=A_n (x_{n-1}^{h_{n-1,n-1} } -x_n^{h_{nn} } ) \\ \end{aligned} \end{aligned}$$

where the dimensionless rate constants are as follows:

10 $$\begin{aligned} A_1&= \alpha _1/X_1^*= \beta _1 X_1^{*h_{11}}/X_1^*, A_2=\beta _1 X_1^{*h_{11}}/X_2^*=\beta _2X_2^{*h_{22}}/X_2^*,\ldots , \nonumber \\ A_i&= \beta _{i-1}X_{i-1}^{*h_{i-1,i-1}}/X_i^*=\beta _i X_i^{*h_{ii} }/X_i^*,\ldots , \nonumber \\ A_n&= \beta _{n-1} X_{n-1}^{*h_{n-1,n-1}}/X_n^*=\beta _n X_n^{*h_{nn}}/X_n^*\end{aligned}$$

Equation (10) provides the following relationship.

11 $$\begin{aligned} X_1^*A_1 =X_2^*A_2 = \cdots =X_i^*A_i =\cdots =X_n^*A_n. \end{aligned}$$

Thus, Eq. (11) provides the following constraint for the linear structure:

12 $$\begin{aligned} A_{i+1} =(X_1^*/X_{i+1}^*)A_1 \quad (i = 1, \ldots , n-1). \end{aligned}$$

In other words, all $A_{i}$ ($i = 2,\ldots , n$) are a function of only $A_{1}$. As a result, Eq. (9) can be expressed in terms of the unknown parameter $A_{1}$. In a linear network structure, the same constraint is also valid for cases with inhibition or activation. The simple case study for a linear structure is discussed in Supplementary Information 1.

#### Branching and Confluent Structures

**Fig. 1:**
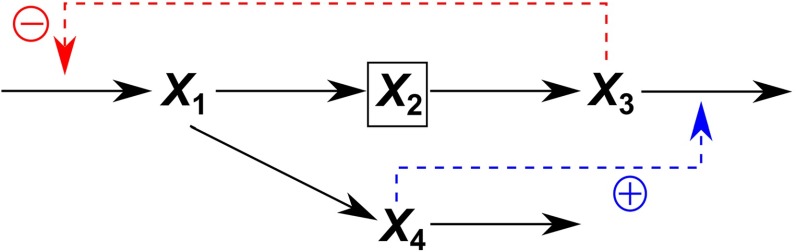
Branched metabolic pathway model with inhibition and activation. $X_{i}$ ($i$=1,...,4) denote metabolites. The square enclosing $X_{2}$ denotes that this metabolite is assumed to be unmeasurable in the “Case where some metabolite concentrations are unmeasurable”

Let us consider a metabolic reaction network where metabolites $X_{1}$ to $X_{m}$ have a linear structure, and $X_{m}$ branches into $X_{m+1}$ and $X_{m+2}$, as in Fig. 1. The S-system equations for this network system are given as

13 $$\begin{aligned} \begin{aligned} \frac{dX_1 }{dt}&=\alpha _1 -\beta _1 X_1^{h_{11} } \\&\quad \vdots \quad \quad \quad \quad \vdots \\ \frac{dX_m }{dt}&=\beta _{m-1} X_{m-1}^{h_{m-1,m-1} } -\beta _m X_m^{h_{m m} } \\ \frac{dX_{m+1} }{dt}&=\alpha _{m+1} X_m^{g_{m+1,m} } -\beta _{m+1} X_{m+1}^{h_{m+1,m+1} } \\ \frac{dX_{m+2} }{dt}&=\alpha _{m+2} X_m^{g_{m+2,m} } -\beta _{m+2} X_{m+2}^{h_{m+2,m+2} } \\&\quad \vdots \quad \quad \quad \quad \vdots \\ \end{aligned} \end{aligned}$$

which give the following relationship at the branching point:

14 $$\begin{aligned} \beta _m X_m^{h_{m m}} =\alpha _{m+1} X_m^{g_{m+1,m} } +\alpha _{m+2} X_m^{g_{m+2,m}}. \end{aligned}$$

Equation (13) can be expressed in dimensionless form as

15 $$\begin{aligned} \begin{aligned} \frac{dx_1 }{dt}&=A_1 (1-x_1^{h_{11} } ) \\&\quad \vdots \quad \quad \quad \quad \;\;\vdots \\ \frac{dx_m }{dt}&=A_m (x_{m-1}^{h_{m-1,m-1} } -x_m^{h_{m m} } ) \\ \frac{dx_{m+1} }{dt}&=A_{m+1} (x_m^{g_{m+1,m} } -x_{m+1}^{h_{m+1,m+1} } ) \\ \frac{dx_{m+2} }{dt}&=A_{m+2} (x_m^{g_{m+2,m} } -x_{m+2}^{h_{m+2,m+2} } ) \\&\quad \vdots \quad \quad \quad \quad \;\;\vdots \\ \end{aligned} \end{aligned}$$

where the dimensionless rate constants are as follows:

16 $$\begin{aligned} A_1&= \alpha _1 /X_1^*=\beta _1 X_1^{*h_{11} } /X_1^*,\ldots ,\nonumber \\ A_m&= \beta _{m-1} X_{m-1}^{*h_{m-1,m-1} } /X_m^*=\beta _m X_m^{*h_{m m} } /X_m^*, \nonumber \\ A_{m+1}&= \alpha _{m+1} X_m^{*g_{m+1,m} } /X_{m+1}^*=\beta _{m+1} X_{m+1}^{h_{m+1,m+1} } /X_{m+1}^*, \nonumber \\ A_{m+2}&= \alpha _{m+2} X_m^{*g_{m+2,m} } /X_{m+2}^*=\beta _{m+2} X_{m+2}^{*h_{m+2,m+2} } /X_{m+2}^*,\ldots \end{aligned}$$

A combination of Eqs. (14) and (16) gives the following relationship:

17 $$\begin{aligned} X_m^*A_m =X_{m+1}^*A_{m+1} +X_{m+2}^*A_{m+2}. \end{aligned}$$

Thus, Eq. (17) indicates that the dimensionless rate constant $A_{m+2}$ can be expressed as a function of the dimensionless rate constant before the branching point, $A_{m}$, and the other dimensionless rate constant at the branching point, $A_{m+1}$, leading to the following constraint:

18 $$\begin{aligned} A_{m+2} =(X_m^*A_m -X_{m+1}^*A_{m+1} )/X_{m+2}^*. \end{aligned}$$

Confluent structures can be treated in a similar manner. The above equations are generalized as follows. When the network has a branching point where the $q$ fluxes of pool $X_{p}$ proceed to the $X_{p+j}$ ($j = 1,\ldots , q$) pools, the following constraint is provided:

19 $$\begin{aligned} X_p^*A_p =\sum _{j=1}^q {X_{p+j}^*A_{p+j}}. \end{aligned}$$

When the network has a confluent structure at which the $q$ fluxes from the $X_{j}$ ($j = 1,\ldots , p$) pools flow into pool $X_{p}$ ($p > q$), the following constraint is obtained:

20 $$\begin{aligned} \sum _{j=1}^q {X_{p-j}^*A_{p-j} } =X_p^*A_p. \end{aligned}$$

### Reason for Assigning Constant Values to Kinetic Orders

It is not easy to determine all the kinetic parameters experimentally in a relatively large-scale system. As the first step of the performance evaluation of the PENDISC method, therefore, the present work assigns an average value of 0.5 or $-0.5$ to the kinetic orders, and determines only the rate constants as unknown parameters by fitting the solutions to S-system equations to the experimental data. This is because the kinetic orders in the power-law equations (transformed from various forms of Michaelis–Menten equations) mostly range from 0 to 1 (or $-1$ and 0 for inhibition), as shown in Fig. S3 (Supplementary Information 3). The determination of all the parameters including the kinetic orders will be discussed in a subsequent study.

The transient behaviors of metabolite concentrations in a network system are mainly governed by the structure of a network. For example, in a linear structure where the metabolites are lined up, variations in the metabolite concentrations are propagated in downstream direction. The magnitudes of these concentration values and the times, at which an increase or decrease in the concentration is reversed, are strongly controlled by the reaction kinetics. In such variations, the rate constants are responsible not only for the magnitudes of metabolite concentrations at each time point but also for shifting reaction curves in the direction of the time axis. On the other hand, the kinetic orders are closely associated with the shapes of the reaction curves, rather than their shift. Thus, the rate constants given in this estimation play an important role in compensating for the differences in the calculated values generated as a result of setting the kinetic orders at 0.5 or $-0.5$.

### Number of Unknown Parameters

For parameter estimation in a relatively large-scale metabolic reaction system, it is very important to reduce the number of unknown parameters as much as possible. This will enhance the rate of convergence, decrease the number of parameter values grouped together, and reduce the estimation time. In an S-system model consisting of $N$ differential equations, the number of unknown parameters is $2N(N + 1)$. This can be reduced to 2$N$ when values of 0.5 or $-0.5$ are assigned to the kinetic orders. It can be further reduced to $N$ when parameter estimation is performed using the time-series data of dimensionless metabolite concentrations. If the network structure is linear, only one parameter becomes unknown. If the structure includes $p$ branching points, then the number of unknown parameters is increased up to $p+1(<N)$.

### Metabolic Reaction Network Models

#### Linear Metabolic Pathway Model with Inhibition

Consider the S-system equations derived from the linear network, as shown in Fig. S1 (Supplementary Information 3). The equations and initial values are given in Supplementary Information 1.

#### Branched Metabolic Pathway Model with Inhibition and Activation

Consider the S-system equations derived from the network structure with a branched metabolic pathway, as shown in Fig. 1 (Voit and Almeida 2004). The equations and initial values are given in Supplementary Information 1.

#### Aspartate-Derived Amino Acid Biosynthesis Model

**Fig. 2:**
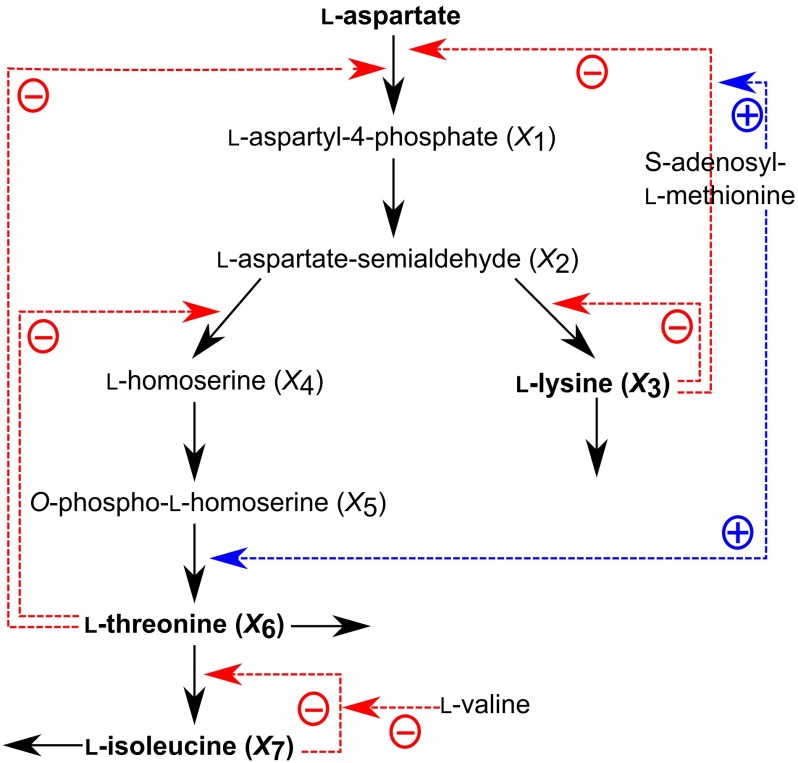
Aspartate-derived amino acid biosynthesis model

The aspartate-derived amino acid biosynthetic pathway in plants is controlled by several feedback inhibitions and activations, and forms a relatively complicated network structure (Fig. 2). The mathematical model for this system consists of seven differential equations with flux expressions in the Michaelis–Menten form (Curien et al. 2009). The kinetic parameters in the expressions have been determined via *in vitro* reconstitution in the model plant *A. thaliana*. The performance of the PENDISC method is investigated by considering the time-series data obtained from in silico calculations as experimental data. The equations and initial values are given in Supplementary Information 1.

### Parameter Estimation

The calculations are performed using a g++ compiler running on Ubuntu Linux 12.04 with Intel(R) Core(TM) i7 CPU 870@2.93 GHz. The Levenberg–Marquardt method is applied to the parameter estimation using a C program adopted from the literature (Press et al. 2002). To enhance the accuracy of numerical derivatives, a highly accurate differentiation method (Shiraishi et al. 2007) is introduced. The $\chi ^{2}$ evaluation function is defined as

21 $$\begin{aligned} \chi ^{2}(\mathbf{A})=\sum _{k=1}^N {\left[ {\frac{X_{i,k} -X_{i,k} (t_k;\mathbf{A})}{\sigma _k }} \right] } ^{2}, \end{aligned}$$

where $X_{i,k}$ is the concentration data of the metabolite $\hbox {X}_{i}$ at the time point $t_{k}$ (or the $k$th data point), $X_{i,k }(t_{k}$; $\mathbf A$) is the calculated concentration of the metabolite $\hbox {X}_{i}$ for the dimensionless rate constant value $A$ at the time point $t_{k}$, and $\sigma _{k}$ is the standard deviation. The calculation is terminated when the relations [$\chi ^{2}(A_{0})-\chi ^{2}(A_{1})$]/ $\chi ^{2}(A_{0}) \le 10^{-8}$ and $\chi ^{2}(A_{0}) \le 10^{-5}$ are satisfied.

## Results and Discussion

### Performance Evaluation of the PENDISC Method

#### Evaluation of the Calculation Algorithm

The performance of the PENDISC method was evaluated using the branched metabolic pathway model with inhibition and activation (shown in Fig. 1). Eleven data points were produced for each evolution, and the initial values of unknown parameters were all set to 5.

**Fig. 3:**
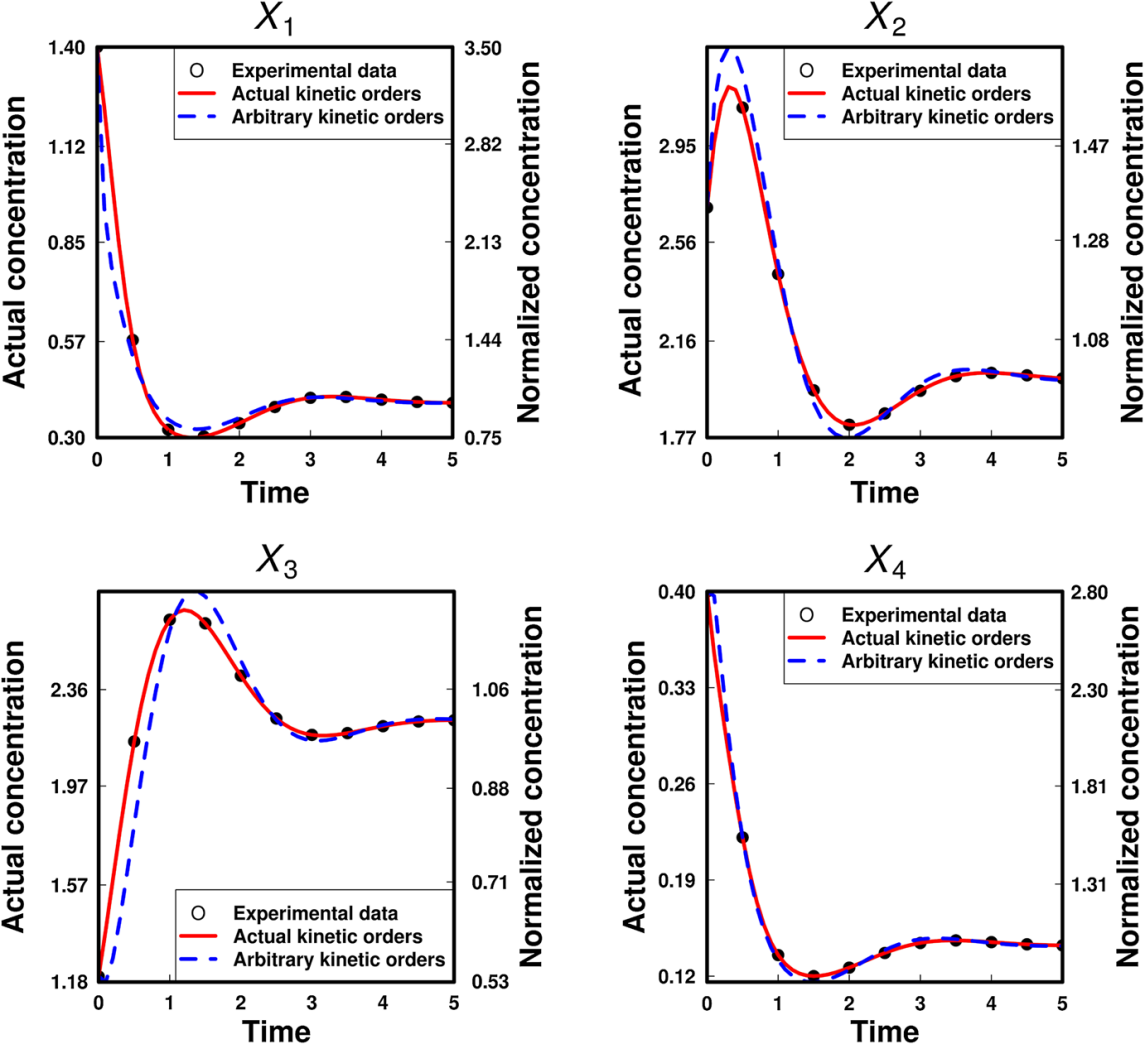
Comparisons of calculated lines based on two estimated parameters and either true kinetic orders (solid lines) or kinetic orders of 0.5 or $-0.5$ (broken lines) with 11 time-series data for each metabolite in a branched metabolic pathway model with inhibition and activation

First, to verify the reliability of model parameters estimated from non-dimensionalized equations, the true values were used for the kinetic orders, and only $$A_{1}$$ and $$A_{4}$$ were estimated as unknown parameters. The result showed that the converged values of $$A_{1}$$ and $$A_{4 }$$ (15.82007531 and 8.85591652) are equivalent to their true values to 10 significant digits. These values of $$A_{1}$$ and $$A_{4}$$ were then used to calculate $$A_{2}$$ and $$A_{3}$$ (2.52077734 and 2.26931039). Consequently, $$\alpha _{1}, \alpha _{2},\alpha _{3}$$, and $$\alpha _{4}$$ were determined as 11.999994655, 7.99999807, 3.00000095, and 1.99999892, respectively, and $$\beta _{1}$$, $$\beta _{2}$$, $$\beta _{3}$$, and $$\beta _{4}$$ were determined as 9.99999555, 2.99999928, 5.00000158, and 5.99999676, respectively. As expected, the values of $$\alpha _{i}$$ and $$\beta _{i}$$ calculated from $$A_{i}$$ ($$i $$= 1, 2, 3, 4) are almost equivalent to their respective true values. The calculated metabolite concentrations are in perfect agreement with the time-series data, as shown in Fig. 3. These results validate the consistency of the calculation algorithm of the PENDISC method.

Second, to evaluate the performance of the PENDISC method, the same parameter estimation was carried out by setting the $A_{i}$ as unknown values and inserting 0.5 or $-0.5$ into the kinetic orders, instead of their true values. The agreement between the calculated results and time-series data is not perfect but satisfactory. This indicates that the solutions to the S-system equations can depict time-transient behaviors analogous to the time-series data, even when arbitrary constant values are used for the kinetic orders, implying that the behavior of the metabolite concentrations is strongly governed by the network structure of a metabolic reaction system. The time courses of the calculated metabolite concentrations are similar to their true ones, although the parameters used for the calculation are different. To prove that the rate constants play a more important role in compensating for the differences in the calculated values, the kinetic orders were set to 0.25, 0.50, 0.75, and 1.00 (to their negative values for inhibition), and parameter estimations were performed in the same manner. The calculated results indicated that the dimensionless rate constants estimated under each condition are rather different, but they can still provide similar patterns for the behaviors of the metabolite concentrations (Fig. S4: Supplementary Information 3).

Third, to analyze the robustness of the PENDISC method, the leave-one-out cross-validation was performed (Fig. S5: Supplementary Information 3). Data points were removed one by one from the 11 time-series data for all metabolites (Fig. S5), and parameters were repeatedly estimated using the remaining 10 time-series data. The results indicated that this method can estimate parameters successfully. Among these removal operations, the performance of the PENDISC method decreased most when the data at $t$ = 0.5 were removed (shown by line P1, mean square error (MSE) = $4.258 \times 10^{-3})$. For comparison, when 11 time-series data were all used, the MSE was $5.293\times 10^{-3}$. Although MSE increased when one data was removed from the time-series data of each metabolite, the calculated result was not significantly different from that for the original data (Table S2: Supplementary Information 2). This implies that the PENDISC method is robust.

Finally, to verify whether this method with fixed kinetic orders is practically applicable, the predictive simulations were performed using the constructed mathematical model. The concentrations of $$X_{1}-X_{4}$$ at a steady state were perturbed by increasing one of the metabolite concentrations by two times of its steady-state value at $$t $$= 0, and the time courses of the metabolite concentrations calculated using the PENDISC model were compared with the time-series data. The results indicated that the PENDISC model provides metabolic behaviors comparable to the exact time-series data (Figure S6: Supplementary Information 3).

#### Effects of the number of time-series data and initial guesses for ${A}_{i}$

The branched metabolic pathway model with inhibition and activation (Fig. 1) was used to investigate the effects of the number of time-series data and initial guesses for $A_{i}$. Experiments were performed using 11, 21, and 51 time-series data, and initial guesses for $A_{i}$ were all set at 5 and 10. Moreover, the following three cases were considered: (1) all four rate constants are estimated; (2) linear pathway constraint is utilized; $A_{3}$ is substituted by $A_{2}X_{2}^{*}/X_{3}^{*}$ and only three rate constants, $A_{1}, A_{2}$, and $A_{4}$, are estimated; and (3) both linear and branching pathway constraints are utilized. $A_{2}$ and $A_{3}$ are substituted by function of $A_{1}$ and $A_{4}$ and only two rate constants, $A_{1}$ and $A_{4}$, are estimated. The calculated results are shown in Fig. S7 (Supplementary Information 3); the dimensionless rate constants determined are listed in Table S3 (Supplementary Information 2), and the calculation times and iteration numbers are given in Table S4 (Supplementary Information 2). The calculated lines are in good agreement with the time-series data, regardless of the number of data points and initial guesses for $A_{i}$, while the calculation time and iteration numbers increase with an increase in the number of data points. This implies that the performance of the PENDISC method depends on data quality rather than data quantity and it is, therefore, possible to shorten the calculation time as a result of reducing the number of time-series data to a limitation where characteristics of the time-transient behaviors of metabolite concentrations are retained.

#### Advantages of Introducing Constraints

The final values of the $\chi ^{2}$ evaluation function, calculation times, and trial numbers in the branched metabolic pathway model with inhibition and activation (Fig. 1) are listed in Table S4 (Supplementary Information 2). It should be noted that the $\chi ^{2}$ evaluation function values for different numbers of time-series data are not directly comparable because it expresses the sum of the squared differences between the metabolite concentration data and their calculated values, and therefore, it increases with an increase in the number of time-series data. Nevertheless, the results pinpoint that a smaller number of unknown parameters tend to decrease the effect of the number of time-series data and the magnitudes of the initial values for $A_{i}$ on the agreement between the calculated and real parameter values. A decrease in the number of unknown parameters also reduces the calculation time and enhances the probability of convergence in the parameter estimation. The PENDISC method enables a substantial reduction in the number of unknown parameters by introducing constraints. Obviously, this advantage is useful in the analysis of relatively large-scale systems.

#### Case Where Some Metabolite Concentrations are Unmeasurable

Any analytical method cannot measure every metabolite concentration in a pathway network. It is, therefore, important to test whether the PENDISC method is applicable to a case where there are some unmeasurable metabolite concentrations in the network.

Consider a case where $X_{2}$ cannot be measured in the branched metabolic pathway model with inhibition and activation (Fig. 1), and each time-series dataset for $X_{1}, X_{3}$, and $X_{4}$ has 11 data points. The constraints were again applied to the differential equations for $X_{1}-X_{4}$, and both $A_{1}$ and $A_{4}$ were set as unknown parameters. Likewise, $A_{2}$ was set as an unknown parameter, because the steady-state concentration of $X_{2}^{*}$ is unknown. Initial values for the dimensionless metabolite concentrations were set as $x_{10 }$= 1.4/0.3996 = 3.5035, $x_{30 }$= 1.2/2.2284 = 0.5385, and $x_{40 }$= 0.4/0.1428 = 2.8011 (see Eqs. (S7) and (S10) in Supplementary Information 1). An arbitrary value must be assigned to $x_{2}$ because both $X_{2}$ and $X_{20}$ are unknown. For this value, we selected the same value as that used in the previous calculation, i.e., $x_{20 }$= 2.7/2.0061 = 1.3459. This is because our aim is to elucidate the extent to which the calculated result of $X_{2}$ deviates from its actual time-series data when once uses the rate constants determined using the value of $A_{2}$ automatically obtained in the estimation of $A_{1}, A_{2}$, and $A_{4}$. The initial values for $A_{1}, A_{2}$, and $A_{4}$ were all set to 5. The parameter estimation was performed by solving the differential equations for $x_{1}-x_{4}$, but the calculated values were fitted only to the evolution of $x_{1},x_{3}$, and $x_{4}$.

**Fig. 4:**
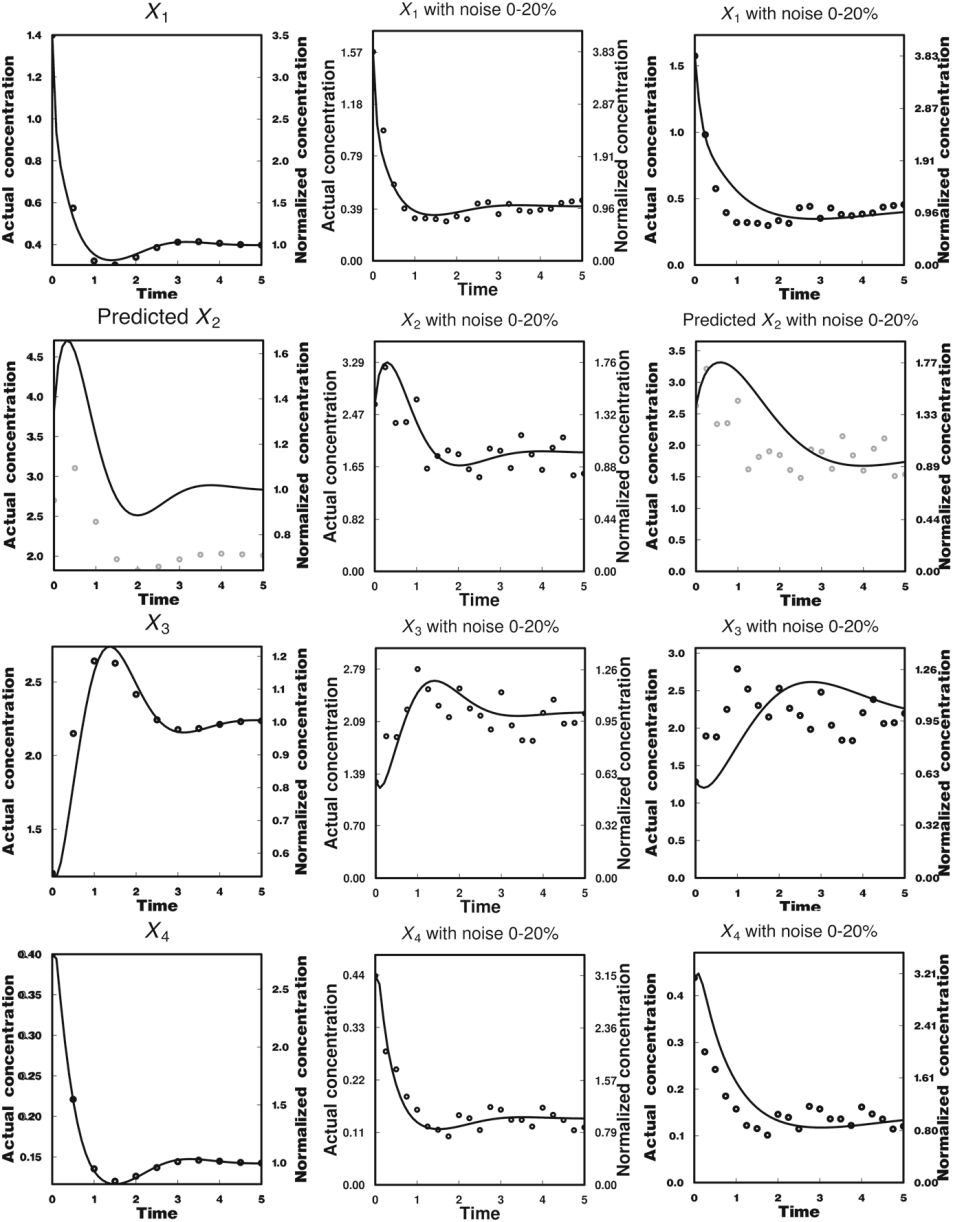
Comparison of calculated lines based on (a) three estimated parameters using 11 time-series data for each metabolite except $X_{2}$ [left column], (b) two estimated parameters using 21 time-series data with up to $\pm$20 % noise for each metabolite [middle column], (c) two estimated parameters using 21 time-series data with up to $\pm$20 % noise for each metabolite except $X_{2}$ [right column] in a branched metabolic pathway model with inhibition and activation

The converged values of $A_{1}, A_{2}$, and $A_{4}$ were 39.62943, 4.38212, and 23.82671, respectively. The relation

22 $$\begin{aligned} A_3 =\frac{A_1 X_1^*-A_4 X_4^*}{X_3^*} \end{aligned}$$

gives a value of 5.57941 for $A_{3}$. In addition, the relation

23 $$\begin{aligned} X_2^*=\frac{A_1 X_1^*-A_4 X_4^*}{A_2 } \end{aligned}$$

provides 2.83721 as the estimated steady-state value of $X_{2}$, i.e., $X^{*}_{2\,\, \mathrm{estimated}}$. This value is 41.43% higher than its true value ($X_{2}^{*}$= 2.00607). The initial value of $X_{2}$ is given as follows: $X_{20 }=x_{20 }\times X^{*}_{2\,\, \mathrm{estimated}}$ = 1.3459 $\times$ 2.83721 = 3.8186. All estimated dimensionless rate constants were used to calculate $\alpha _{i}$ and $\beta _{i}$. The results are listed in Table S5 (Supplementary Information 2). The metabolite concentrations calculated using the rate constants are compared with their respective time-series data in the left column of Fig. 4. The calculated lines for $X_{1}, X_{3}$, and $X_{4}$ are in good agreement with their respective time-series data. On the other hand, the calculated line for $X_{2}$ is not entirely in good agreement with its original time-series data, because the value of $X^{*}_{2\,\, \mathrm{estimated}}$ was markedly different from its original value. Nevertheless, it is interesting to note that the variation in the calculated values of $X_{2}$ is very similar to that in the original data.

When a value of the same order of magnitude as the initial values (Fig. S8: Supplementary Information 3) of other dimensionless metabolite concentrations was assigned to $x_{20}$, changes occurred not only for $A_{2}$ and $X^{*}_{2\,\, \mathrm{estimated}}$, but also for $A_{1}, A_{3}$, and $A_{4}$. This, in turn, changed the calculated line for $X_{2}$, whereas those for $X_{1}, X_{3}$, and $X_{4}$ were almost the same as before. It is, therefore, deduced that the PENDISC method enables us to construct a mathematical model, even when metabolite concentrations in a network are partially unmeasurable. It should be noted that the calculated lines are not identical to the true time-series concentrations because the steady-state concentrations are estimated using measured data containing a large number of errors. Fortunately, it seems that the calculated line successfully generates the behavior of the unmeasurable metabolite concentrations. This is because the metabolic behaviors are governed mainly by the network structure, and the time courses of metabolite concentrations tend to move or shift in different magnitudes when parameter values are different. However, to predict the metabolic behaviors more accurately, one may make efforts to measure the steady-state values of the unmeasurable metabolite concentrations by introducing other different analytical instruments.

#### Treatment of Time-Series Data with Noise

Measured metabolite concentrations usually contain biological variations and analytical errors. The effect of noise on the probability of convergence was investigated using the branched metabolic pathway model with inhibition and activation (Fig. 1). Twenty-one time-series data with random noise of up to $\pm$20% were produced for each metabolite concentration (Fig. 4, middle column). The values of the time-series data in the neighborhood of the steady-state condition (the last five data) were averaged to obtain the experimental steady-state concentration for each metabolite ($X_{1}^{*}$= 0.41114, $X_{2}^{*}$= 1.86921, $X_{3}^{*}$= 2.20575, $X_{4}^{*}$= 0.13928). Again, $A_{1}$ and $A_{4}$ were set as unknown parameters, and the initial guesses for dimensionless rate constants were all set to 5. The parameter estimation provided 33.16106 and 32.19971 of $A_{1}$ and $A_{4}$, respectively. These values and those for $X_{1}^{*}$, $X_{2}^{*}, X_{3}^{*}$, and $X_{4}^{*}$ were inserted into the relevant equations to obtain $A_{2}$ = 4.89464 and $A_{3}$ = 4.14785. All the values were further used to calculate $\alpha _{i}$ and $\beta _{i}$. The time courses of metabolite concentrations calculated using the rate constants determined are compared with the true time-series data in Fig. 4 (middle column). The calculated lines are in pretty agreement with the time-series data, indicating that the PENDISC method can also handle noisy time-series data successfully.

#### Case for Time-Series Data Containing Both Noisy and Unmeasurable Metabolite Concentrations

We further consider case where metabolite concentrations contain biological variations and analytical errors, and some of the metabolite concentrations are unmeasurable in the branched metabolic pathway model with inhibition and activation. Twenty-one time-series data with random noise of up to $\pm$20% were produced for each metabolite concentration, and the concentration of $X_{2}$ was assumed to be unmeasurable (Fig. 4, right column). Parameters were estimated using the same algorithm, initial values, and steady-state values as described in the previous sections. The values of $A_{1}$ and $A_{4}$ were determined as 16.35916 and 14.07396, respectively, and the values of $A_{2}$ and $A_{3}$ were then calculated as 2.54958 and 2.16059, respectively. The calculated lines using the determined values were in reasonable agreement with the time-series data with noise, and the time-transient behaviors of the unmeasurable metabolite concentration were predicted successfully.

The results indicate that the PENDISC method has high potential for handling both noisy and unmeasurable metabolite concentration data. The method does not require us to use true parameter values in the first step of parameter estimation and provides calculated results close to the behavior of the time-series data of metabolite concentrations, which will allow us to estimate better parameter values and then construct a useful mathematical model to analyze metabolic reaction systems.

### Application of the PENDISC Method to an Actual Metabolic System

The aspartate-derived amino acid biosynthesis illustrated in Fig. 2 is used as a practical application of the PENDISC method. It is assumed that the system is initially at a steady-state value, and the l-aspartyl-4-phosphate concentration, $X_{1}$, is increased by two times of its steady-state value at $t$= 0. As a result, all the metabolite concentrations start to change and finally return to their original steady-state values. Twenty-one time-series data were produced in silico for each metabolite concentration. S-system equations were derived from Fig. 2, and the kinetic orders in these equations were set to 0.5 or $-0.5$. The resulting equations were rearranged using the relevant constraints. Consequently, the number of unknown dimensionless rate constants was reduced from seven to two.

**Fig. 5:**
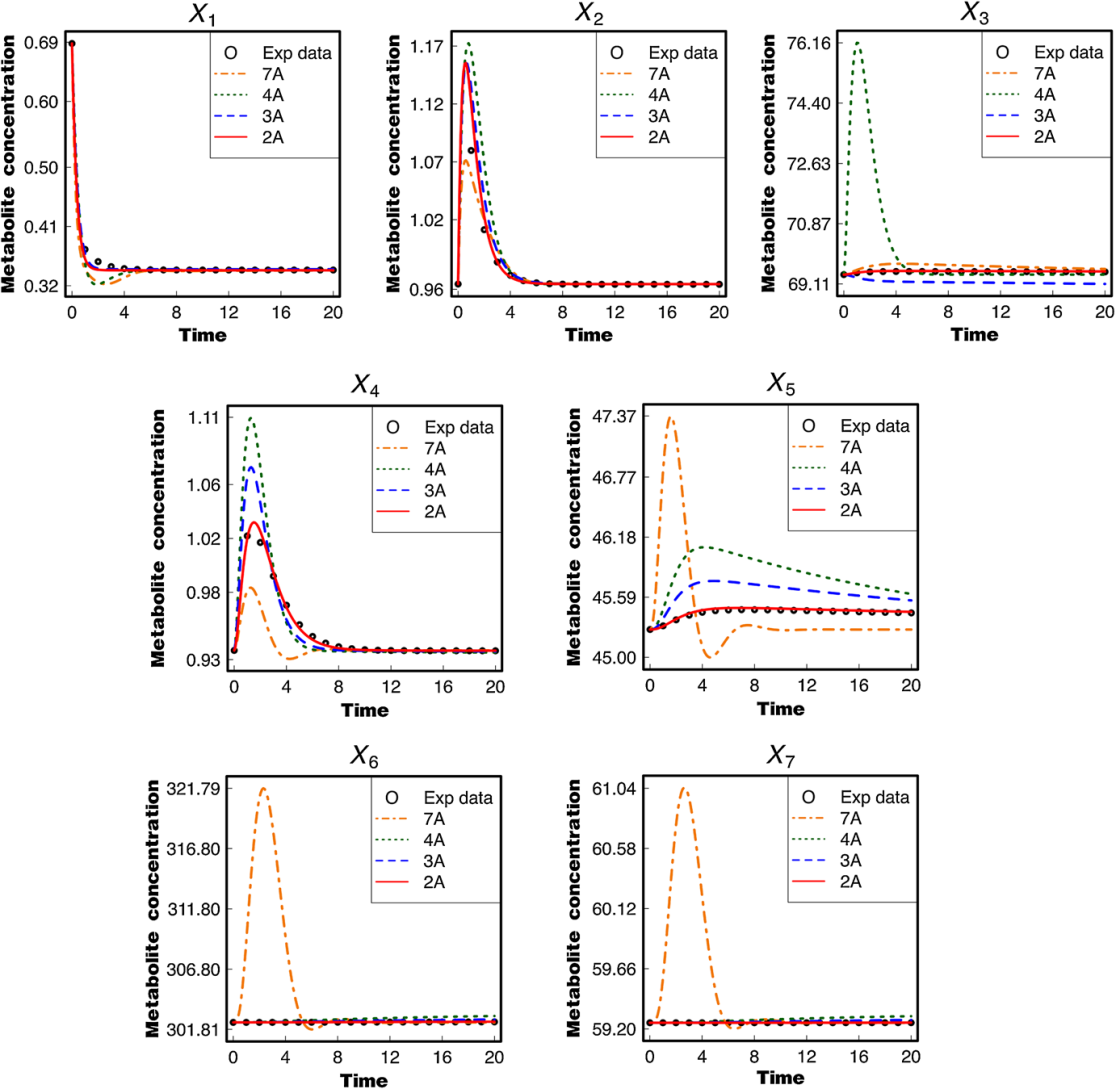
Comparison of calculated lines based on two, three, four, and seven estimated parameters (represented by the symbols “2A,” “3A,” “4A,” and “7A,” respectively) with 21 time-series data for each metabolite in an aspartate-derived amino acid formation model. The initial guesses were all set to 5

Figure 5 compares the metabolite concentrations calculated using two, three, four, and seven estimated rate constants (referred to as 2A, 3A, 4A, and 7A, respectively) with their respective time-series data. The concentrations of l-aspartyl-4-phosphate ($X_{1}$), l-aspartate-semialdehyde ($X_{2}$), and l-homoserine ($X_{4}$) change rapidly, whereas those of l-lysine ($X_{3}$), $O$-phospho-l-homoserine ($X_{5}$), l-threonine ($X_{6}$), and l-isoleucine ($X_{7}$) change very slowly. This is because the stiffness ratio (defined as the ratio of the maximum and minimum absolute values of real parts of eigenvalues) is equal to $1.757 \times 10^{3} (=14.826995/8.4379835 \times 10^{-3}$) which means that the system is mildly stiff. The calculated results are not in good agreement with the data in the cases of four and seven estimated parameters, whereas the situation is improved in the case of three estimated parameters. Interestingly, the calculated lines for all metabolites, including very slowly changing $X_{6}$ and $X_{7}$, were in very good agreement with the time-series data in the case of two estimated parameters (Fig. S9: Supplementary Information 3); the estimated parameter values for this case are listed in Table S6 (Supplementary Information 2). This implies that introducing the relevant constraints allow us to reduce the number of parameters to be estimated, resulting in an increase in fitting performance and also decreases in both iteration number and calculation time. These advantages would make it easier to construct a mathematical model in a relatively large-scale system.

A combination of decoupling and grid methods also allows us to roughly estimate parameters in the construction of a coarse model (Iwata et al. 2013). It reduces the number of parameters greatly, which makes it easy to estimate the parameters, but requires us to calculate the time rates of change of the metabolite concentrations (i.e., slopes) for the parameter estimation. Since this calculation requires us to carry out data smoothing while handling noisy time-series data, the result of parameter estimation may be affected by which smoothing method is chosen. On the other hand, although the PENDISC method uses averaged values for the kinetic orders, it does not need the slopes. Nevertheless, it can offer satisfactory behaviors for the metabolite concentrations. It is also potentially applicable to the case where true values are necessary for the kinetic orders, since the number of rate constants to be estimated is halved by non-dimensionalization of the S-system equations, which, in turn, increases the probability of convergence. The number of rate constants can be further reduced by the use of the constraints. For example, in a linear metabolic pathway, the estimation of only one rate constant and kinetic order makes it possible to calculate the evolution of every metabolite concentration. The extension of the PENDISC method to the parameter estimation including kinetic order values will be discussed in a subsequent study.

## Conclusions

The present study proposed a simple method for constructing a mathematical model for a metabolic reaction network, named the PENDISC method, in which the number of parameters to be estimated is reduced by use of non-dimensionalized S-system equations with constraints, and then investigated its performance using three mathematical models. As a result, the following conclusions are derived:

1. The relevant constraints produced from a network structure are useful for reducing the number of dimensionless rate constants significantly.
2. Even when the values of 0.5 or $-0.5$ are used for the kinetic orders, the resulting S-system model can successfully exhibit the dynamic behaviors of metabolite concentrations analogous to the evolution of true ones.
3. As the number of time-series data decreases, the agreement of the calculated result with the time-series data increases and the iteration number exponentially decreases, which results in a decrease in the calculation time.
4. A significant reduction in the number of dimensionless rate constants as a result of introducing the relevant constraints improves the chance of convergence.
5. The PENDISC method can construct a mathematical model even when some of metabolite concentrations in a network are unmeasurable, and the time-series data include noise.

## Electronic supplementary material

Below is the link to the electronic supplementary material.

Supplementary material 1 (pdf 2233 KB)
