# Supplementary material for: PENDISC: A Simple Method for Constructing a Mathematical Model from Time-Series Data of Metabolite Concentrations
Source: Bull Math Biol. 2014 May 7;76(6):1333–51. doi: 10.1007/s11538-014-9960-8 (PMC4048473; doi:10.1007/s11538-014-9960-8)
Supplement: Supplementary file 1 — Supplementary material 1 (pdf 2233 KB) [file 11538_2014_9960_MOESM1_ESM.pdf]

Supplementary Information: PENDISC: A Simple Method for Constructing a Mathematical Model from Time-Series Data of Metabolite Concentrations

Author: Kansuporn Sriyudthsak[1,2,3], Michio Iwata[4], Masami Yokota Hirai[1,2,3,*], and Fumihide Shiraishi[4,*]
Affiliation: [1]RIKEN Plant Science Center, Yokohama 230-0045, Japan
[2]RIKEN Center for Sustainable Resource Science, Yokohama 230-0045, Japan
[3]JST, CREST, Kawaguchi 332-0012, Japan
[4]Graduate School of Bioresource and Bioenvironmental Sciences, Kyushu University, Fukuoka 812-8581, Japan

*Authors to whom correspondence should be addressed.
Masami Yokota Hirai, Ph.D.
Metabolic Systems Research Team
RIKEN Center for Sustainable Resource Science
1-7-22 Suehiro-cho, Tsurumi-ku, Yokohama, Kanagawa 230-0045 Japan
Email: masami.hirai@riken.jp

Fumihide Shiraishi, Ph.D.
Graduate School of Bioresource and Bioenvironmental Sciences
Kyushu University
Hakozaki, Fukuoka 812-8581 Japan
Email: fumishira@brs.kyushu-u.ac.jp

Supplementary Information 1: Additional information

1.1 Simple case study for linear metabolic pathway model with inhibition

Let us consider the following S-system equations that are derived from the linear network shown in Fig. S1.

$$\begin{aligned}\frac{dX_1}{dt} &= 12X_3^{-0.8} - 10X_1^{0.5} \\ \frac{dX_2}{dt} &= 10X_1^{0.5} - 3X_2^{0.75} \\ \frac{dX_3}{dt} &= 3X_2^{0.75} - 5X_3^{0.5}\end{aligned} \tag{S1}$$

The initial values for the metabolite concentrations are chosen as X_{10} = 1.4, X_{20} = 2.7, and X_{30} = 1.2. The steady-state metabolite concentrations are as follows; X_1^*= 0.4902, X_2^* = 3.0958, and X_3^* =1.9610. In an actual system, these concentrations must be determined from measured values. Firstly, values of 0.5 or –0.5 are assigned to the kinetic orders in Eq. (S1) to obtain the following equations.

$$\begin{aligned}\frac{dX_1}{dt} &= \alpha_1 X_3^{-0.5} - \beta_1 X_1^{0.5} \\ \frac{dX_2}{dt} &= \beta_1 X_1^{0.5} - \beta_2 X_2^{0.5} \\ \frac{dX_3}{dt} &= \beta_2 X_2^{0.5} - \beta_3 X_3^{0.5}\end{aligned} \tag{S2}$$

Secondly, Eq. (S2) is non-dimensionalized using steady-state concentrations, followed by applying the constraints:

$$\begin{aligned}A_2 &= (X_1^* / X_2^*) A_1 \\ A_3 &= (X_1^* / X_3^*) A_1\end{aligned} \tag{S3}$$

which mathematical operation leads to

$$\frac{dx_1}{dt} = A_1(x_3^{-0.5} - x_1^{0.5})$$
$$\frac{dx_2}{dt} = A_2(x_1^{0.5} - x_2^{0.5}) = A_1\frac{X_1^*}{X_2^*}(x_1^{0.5} - x_2^{0.5}) \tag{S4}$$
$$\frac{dx_3}{dt} = A_3(x_2^{0.5} - x_3^{0.5}) = A_1\frac{X_1^*}{X_3^*}(x_2^{0.5} - x_3^{0.5})$$

where

$$A_1 = \alpha_1 X_3^{*-0.5} = \beta_1 X_1^{*0.5}, \quad A_2 = \beta_1 X_1^{*0.5} = \beta_2 X_2^{*0.5}, \quad A_3 = \beta_2 X_2^{*0.5} = \beta_3 X_3^{*0.5}. \tag{S5}$$

Lastly, the value of A_1 is determined by a nonlinear least-squares method so as to fit the solution to Eq. (S4) to the time-series data.

In general, the chance of the parameter estimation converging increases as the number of unknown parameters decreases. Fortunately, the PENDISC method can markedly reduce the number of unknown parameters using the constraints derived from the structure of the network. To confirm the validity of this operation, the parameter estimation in the linear metabolic pathway model with inhibition (Fig. S1) was performed for two cases, one in which the values of A_1, A_2, and A_3 are unknown, and a second in which only the value of A_1 is unknown. Figure S2 shows a comparison of the results obtained using the estimated rate constants with the time-series data of metabolite concentrations; the left vertical axis shows the actual metabolite concentration, and the right vertical axis shows the dimensionless metabolite concentration. It should be noted that these two metabolite concentrations are different in magnitude, but vary in the same manner. Eleven concentration data points were used for each metabolite, and the initial values of A_i were all set to 5. The rate constants determined for different numbers of unknown parameters are listed in Table S1 (Supplementary Information 2). The agreement between the calculated results and the time-series data is reasonable, but not perfect. This is because the kinetic orders in the S-system equations were set to 0.5 or –0.5. In every case, the calculated lines successfully exhibit time-transient behaviors analogous to the time-series data. The agreement is higher in the case of three unknown rate constants than in the case of one unknown rate constant. This is because the former case has more flexibility for changing the shape of the calculated line, as there are more adjustable parameters. However, it should be noted that when three dimensionless rate constants are unknown, the relevant equations do not satisfy the constraints. In theory, the calculated result can satisfy the

constraints only when A_1 is unknown and other dimensionless rate constants are expressed as a function of A_1. The calculation time was 6.34 s for the case of three unknown parameters, and this shortened to 4.67 s for the case of one unknown parameter.

Evidently, a reduction in the number of unknown parameters is the major advantage of the PENDISC method. When the pathway is linear, all the relevant differential equations are expressed in terms of the dimensionless rate constant of the first metabolite (A_1), regardless of the total number of metabolites. If the steady-state values and time-series data are available for all metabolites, the rate constants α_i and β_i (i = 1, 2, ...,n) can be obtained by simply determining A_1 in order to fit the calculated values to the time-series data for all metabolite concentrations.

1.2 Equations for branched pathway model with inhibition and activation

Let us consider the following S-system equations derived from the network structure with a branched pathway shown in Fig. 2 in the manuscript (Voit and Almeida, 2004).

$$\begin{aligned}
\frac{dX_1}{dt} &= 12X_3^{-0.8} - 10X_1^{0.5} \\
\frac{dX_2}{dt} &= 8X_1^{0.5} - 3X_2^{0.75} \\
\frac{dX_3}{dt} &= 3X_2^{0.75} - 5X_3^{0.5}X_4^{0.2} \\
\frac{dX_4}{dt} &= 2X_1^{0.5} - 6X_4^{0.8}
\end{aligned} \tag{S6}$$

The initial values for the metabolite concentrations are chosen as

$$X_{10} = 1.4, X_{20} = 2.7, X_{30} = 1.2, \text{ and } X_{40} = 0.4. \tag{S7}$$

The steady-state concentrations are as follows; X_1*= 0.3996, X_2^* = 2.0061, X_3^* = 2.2284, and X_4^* = 0.1428. Firstly, values of 0.5 or –0.5 are assigned to the kinetic orders in Eq. (S6) to obtain the following equations.

$$
\begin{aligned}
\frac{dX_1}{dt} &= \alpha_1 X_3^{-0.5} - \beta_1 X_1^{0.5} \\
\frac{dX_2}{dt} &= \alpha_2 X_1^{0.5} - \beta_2 X_2^{0.5} \\
\frac{dX_3}{dt} &= \beta_2 X_2^{0.5} - \beta_3 X_3^{0.5} X_4^{0.5} \\
\frac{dX_4}{dt} &= \alpha_4 X_1^{0.5} - \beta_4 X_4^{0.5}
\end{aligned}
\tag{S8}
$$

Secondly, Eq. (S8) is non-dimensionalized using steady-state concentrations, followed by applying the constraints:

$$
A_2 = (X_1^* A_1 - X_4^* A_4) / X_2^*, \quad A_3 = (X_1^* A_1 - X_4^* A_4) / X_3^* . \tag{S9}
$$

which leads to

$$
\begin{aligned}
\frac{dx_1}{dt} &= A_1 (x_3^{-0.5} - x_1^{0.5}) \\
\frac{dx_2}{dt} &= A_2 (x_1^{0.5} - x_2^{0.5}) = \frac{A_1 X_1^* - A_4 X_4^*}{X_2^*} (x_1^{0.5} - x_2^{0.5}) \\
\frac{dx_3}{dt} &= A_3 (x_2^{0.5} - x_3^{0.5} x_4^{0.5}) = \frac{A_1 X_1^* - A_4 X_4^*}{X_3^*} (x_2^{0.5} - x_3^{0.5} x_4^{0.5}) \\
\frac{dx_4}{dt} &= A_4 (x_1^{0.5} - x_4^{0.5})
\end{aligned}
\tag{S10}
$$

with the following dimensionless rate constants.

$$
\begin{aligned}
A_1 &= \alpha_1 X_3^{*-0.5} / X_1^* = \beta_1 X_1^{*0.5} / X_1^*, \\
A_2 &= \alpha_2 X_1^{*0.5} / X_2^* = \beta_2 X_2^{*0.5} / X_2^*, \\
A_3 &= \beta_2 X_2^{*0.5} / X_3^* = \beta_3 X_3^{*0.5} X_4^{*0.5} / X_3^*, \\
A_4 &= \alpha_4 X_1^{*0.5} / X_4^* = \beta_4 X_4^{*0.5} / X_4^*
\end{aligned}
\tag{S11}
$$

Lastly, two unknown parameters included in Eq. (S10), A_1 and A_4, are determined by a nonlinear least-squares method so as to fit the solution to Eq. (S10) to the time-series data.

1.3 Equations for aspartate-derived amino acid biosynthesis model

The aspartate-derived amino acid biosynthesis in plants is controlled by several feedback inhibitions and activations and forms a relatively complicated network

structure shown in Fig. 3 in the manuscript. The mathematical model for this system consists of seven differential equations with the flux expressions in Michaelis-Menten form (Curien, et al., 2009). The kinetic parameters in the expressions have been determined by *in vitro* measurement of metabolite concentrations in the plant model, *Arabidopsis*. In the following, we investigated the performance of the PENDISC method by regarding the time-series data in *in silico* calculation as experimental data.

When the mathematical model is transformed into S-system equations and a value of 0.5 or –0.5 is assigned to the kinetic orders, the following equations are obtained.

$$\begin{aligned}
\frac{dX_1}{dt} &= \alpha_1 X_3^{-0.5} X_6^{-0.5} - \beta_1 X_1^{0.5} \\
\frac{dX_2}{dt} &= \beta_1 X_1^{0.5} - \beta_2 X_2^{0.5} X_3^{-0.5} X_6^{-0.5} \\
\frac{dX_3}{dt} &= \alpha_3 X_2^{0.5} X_3^{-0.5} - \beta_3 X_3^{0.5} \\
\frac{dX_4}{dt} &= \alpha_4 X_2^{0.5} X_6^{-0.5} - \beta_4 X_4^{0.5} \\
\frac{dX_5}{dt} &= \beta_4 X_4^{0.5} - \beta_5 X_5^{0.5} \\
\frac{dX_6}{dt} &= \beta_5 X_5^{0.5} - \beta_6 X_6^{0.5} X_7^{-0.5} \\
\frac{dX_7}{dt} &= \beta_6 X_6^{0.5} X_7^{-0.5} - \beta_7 X_7^{0.5}
\end{aligned} \qquad \text{(S12)}$$

The initial values for the metabolite concentrations are chosen as

$$\begin{aligned}
&X_{10} = 0.688, X_{20} = 0.9645, X_{30} = 69.3809, X_{40} = 0.9368, \\
&X_{50} = 45.2733, X_{60} = 302.3907, X_{70} = 59.2472
\end{aligned} \qquad \text{(S13)}$$

The model provides the steady-state concentrations as follows; $X_1^* = 0.3440$, $X_2^* = 0.9645$, $X_3^* = 69.3809$, $X_4^* = 0.9368$, $X_5^* = 45.2733$ $X_6^* = 302.3907$, and $X_7^* = 59.2472$. Eq. (S12) is routinely non-dimensionalized using these values, followed by applying the constraints:

$$\begin{aligned}
&A_2 = (X_1^* / X_2^*) A_1, \quad A_3 = (X_2^* A_2 - X_4^* A_4) / X_3^* = (X_1^* A_1 - X_4^* A_4) / X_3^* \\
&A_5 = (X_4^* / X_5^*) A_4, \quad A_6 = (X_4^* / X_6^*) A_4, \quad A_7 = (X_4^* / X_7^*) A_4
\end{aligned} \qquad \text{(S14)}$$

which leads to

$$
\begin{aligned}
\frac{dx_1}{dt} &= A_1\left(x_3^{-0.5}x_6^{-0.5} - x_1^{0.5}\right) \\
\frac{dx_2}{dt} &= A_2\left(x_1^{0.5} - x_2^{0.5}x_3^{-0.5}x_6^{-0.5}\right) = A_1\frac{X_1^*}{X_2^*}\left(x_1^{0.5} - x_2^{0.5}x_3^{-0.5}x_6^{-0.5}\right) \\
\frac{dx_3}{dt} &= A_3\left(x_2^{0.5}x_3^{-0.5} - x_3^{0.5}\right) = \frac{X_1^*A_1 - X_4^*A_4}{X_3^*}\left(x_2^{0.5}x_3^{-0.5} - x_3^{0.5}\right) \\
\frac{dx_4}{dt} &= A_4\left(x_2^{0.5}x_6^{-0.5} - x_4^{0.5}\right) \\
\frac{dx_5}{dt} &= A_5\left(x_4^{0.5} - x_5^{0.5}\right) = A_4\frac{X_4^*}{X_5^*}\left(x_4^{0.5} - x_5^{0.5}\right) \\
\frac{dx_6}{dt} &= A_6\left(x_5^{0.5} - x_6^{0.5}x_7^{-0.5}\right) = A_4\frac{X_4^*}{X_6^*}\left(x_5^{0.5} - x_6^{0.5}x_7^{-0.5}\right) \\
\frac{dx_7}{dt} &= A_7\left(x_6^{0.5}x_7^{-0.5} - x_7^{0.5}\right) = A_4\frac{X_4^*}{X_7^*}\left(x_6^{0.5}x_7^{-0.5} - x_7^{0.5}\right)
\end{aligned}
\tag{S15}
$$

with the following dimensionless rate constants.

$$
\begin{aligned}
A_1 &= \alpha_1 X_3^{*-0.5}X_6^{*-0.5} / X_1^* = \beta_1 X_1^{*0.5} / X_1^*, \\
A_2 &= \beta_1 X_1^{*0.5} / X_2^* = \beta_2 X_2^{*0.5}X_3^{*-0.5}X_6^{*-0.5} / X_2^*, \\
A_3 &= \alpha_3 X_2^{*0.5}X_3^{*-0.5} / X_3^* = \beta_3 X_3^{*0.5} / X_3^*, \\
A_4 &= \alpha_4 X_2^{*0.5}X_6^{*-0.5} / X_4^* = \beta_4 X_4^{*0.5} / X_4^*, \\
A_5 &= \beta_4 X_4^{*0.5} / X_5^* = \beta_5 X_5^{*0.5} / X_5^*, \\
A_6 &= \beta_5 X_5^{*0.5} / X_6^* = \beta_6 X_6^{*0.5}X_7^{*-0.5} / X_6^*, \\
A_7 &= \beta_6 X_6^{*0.5}X_7^{*-0.5} / X_7^* = \beta_7 X_7^{*0.5} / X_7^*
\end{aligned}
\tag{S16}
$$

Eq. (S15) includes two unknown parameters, A_1 and A_4. This is because the present network model possesses one branching point (p=1) and therefore, the number of parameters becomes equal to 2 by $1+p$. Lastly, A_1 and A_4 are determined by a nonlinear least-squares method so as to fit the solution to Eq. (S15) to the time-series data.

Supplementary Information 2: Additional tables

Table S1 Parameters estimated in a linear metabolic pathway model with inhibition

	Number of unknown parameters	
	3	1
A_1	10.91306	15.49400
A_2	2.56352	2.45355
A_3	5.29521	3.87350
α_1	7.49190	10.63674
α_2	11.33469	10.84847
α_3	5.90152	4.31702
β_1	7.64102	10.84847
β_2	4.51051	4.31702
β_3	7.41511	5.42424

Table S2 Mean square error (MSE) from leave-one-out cross validation in a branched pathway model with inhibition and activation

Cases	Mean Square Errors (MSE)
Original data	4.257729394011472e-03
Original data without P1 (leave data at t=0.5 out)	5.292934493602961e-03
Original data without P2 (leave data at t=1.0 out)	5.049823299379336e-03
Original data without P3 (leave data at t=1.5 out)	4.192725465875488e-03
Original data without P4 (leave data at t=2.0 out)	4.171368192927288e-03
Original data without P5 (leave data at t=2.5 out)	4.239005265752483e-03
Original data without P6 (leave data at t=3.0 out)	4.263600386371204e-03
Original data without P7 (leave data at t=3.5 out)	4.251004518991907e-03
Original data without P8 (leave data at t=4.0 out)	4.251371094692189e-03
Original data without P9(leave data at t=4.5 out)	4.256641447206231e-03
Original data without P10 (leave data at t=5.0 out)	4.257691739470663e-03

Table S3 Dimensionless rate constants estimated under different conditions in a branched pathway model with inhibition and activation

	Number of data points		
	11	21	51
4AI5-A_1 *)	37.40380	21.30459	20.14537
4AI5-A_2	4.43753	5.12628	5.19780
4AI5-A_3	5.82703	4.20929	3.96235
4AI5-A_4	23.65609	136.04615	5019.22171
4A I10-A_1	37.40380	21.30459	20.14534
4A I10-A_2	4.43753	5.12628	5.19825
4A I10-A_3	5.82703	4.20929	3.96243
4A I10-A_4	23.65609	136.04614	5019.20585
3A I5-A_1	28.92633	22.04351	20.45549
3A I5-A_2	5.12280	4.92922	4.73220
3A I5-A_3	4.61176	4.43749	4.26013
3A I5-A_4	36.46860	95.18853	4938.25547
3A I10-A_1	28.92633	22.04351	20.46039
3A I10-A_2	5.12280	4.92922	4.73178
3A I10-A_3	4.61176	4.43749	4.25974
3A I10-A_4	36.46860	95.18853	4938.42454
2A I5-A_1	36.12924	35.72481	35.25168
2A I5-A_2	4.96854	4.74889	4.71687
2A I5-A_3	4.47289	4.27515	4.24633
2A I5-A_4	31.30257	33.25723	32.38298
2A I10-A_1	36.12924	35.72481	35.25168
2A I10-A_2	4.96854	4.74889	4.71687
2A I10-A_3	4.47289	4.27515	4.24633
2A I10-A_4	31.30257	33.25723	32.38297

*) The numerical values before the character "A" and after the character "I" indicate the number of unknown parameters and the value used as the initial guesses, respectively.

Table S4 χ^2, calculation times, and iterations with different conditions in a branched pathway model with inhibition and activation

	Number of data points		
	11	21	51
4A Initial5*) - χ^2	0.05898	0.30021	0.93860
Calculation time (s)	9.51	813.06	35874.42
Iteration (times)	16	733	4854
4A Initial10 - χ^2	0.05898	0.30021	0.93860
Calculation time (s)	9.86	55.15	34994.06
Iteration (times)	19	35	4780
3A Initial5 - χ^2	0.06925	0.30321	0.97750
Calculation time (s)	9.53	34.06	66353.40
Iteration (times)	21	16	8768
3A Initial10 - χ^2	0.06925	0.30321	0.97750
Calculation time (s)	7.12	31.82	64585.90
Iteration (times)	13	17	9074
2A Initial5 - χ^2	0.07441	0.43083	1.56563
Calculation time (s)	15.48	69.55	556.67
Iteration (times)	37	45	50
2A Initial10 - χ^2	0.07441	0.43083	1.56563
Calculation time (s)	13.51	66.35	555.75
Iteration (times)	30	41	49

*) The numerical value before the character "A" indicates the number of unknown parameters and that after the word "Initial" represents the value used as the initial guesses

Table S5 Rate constants estimated in a branched pathway model with inhibition and activation in a case of unmeasurable X_2.

X_i	α_i	β_i
X_1	23.63709	25.05008
X_2	19.66911	7.38125
X_3	7.38125	22.04390
X_4	5.38097	9.00239

Table S6 Parameters estimated in an aspartate-derived amino acid biosynthesis model

X_i	A_i	α_i	β_i
X_1	6.44094	320.96584	3.77791
X_2	2.29758	3.77791	326.82616
X_3	0.01045	6.15125	0.08707
X_4	1.59116	26.39523	1.54010
X_5	0.03293	1.54010	0.22155
X_6	0.00493	0.22155	0.65983
X_7	0.02516	0.65983	0.19366

Supplementary Information 3: Additional figures

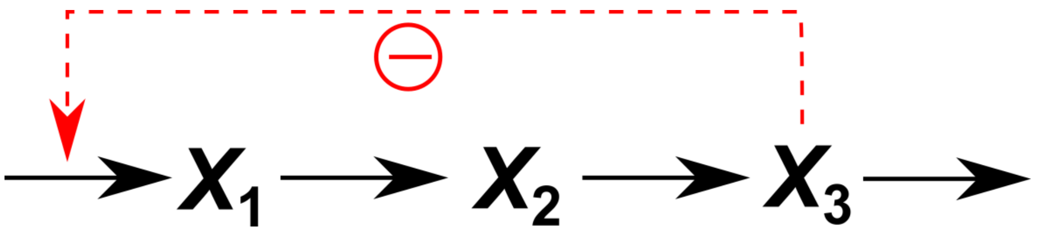

Fig. S1 Linear metabolic pathway model with inhibition

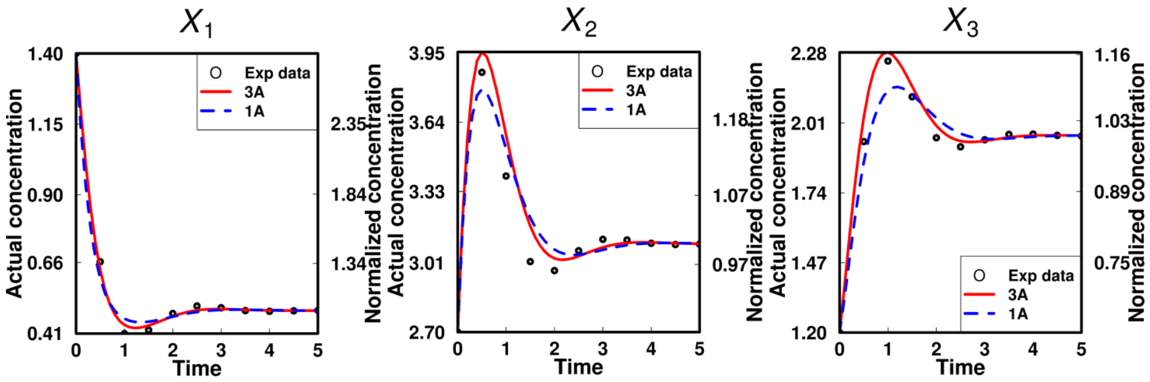

Fig S2 Comparisons of the calculated lines based on three estimated parameters (3A–solid lines) and one parameter (1A–broken lines) with 11 time-series data for each metabolite in a linear metabolic pathway model with inhibition. The left vertical axis shows the actual metabolite concentration, whereas the right vertical axis shows the dimensionless metabolite concentration.

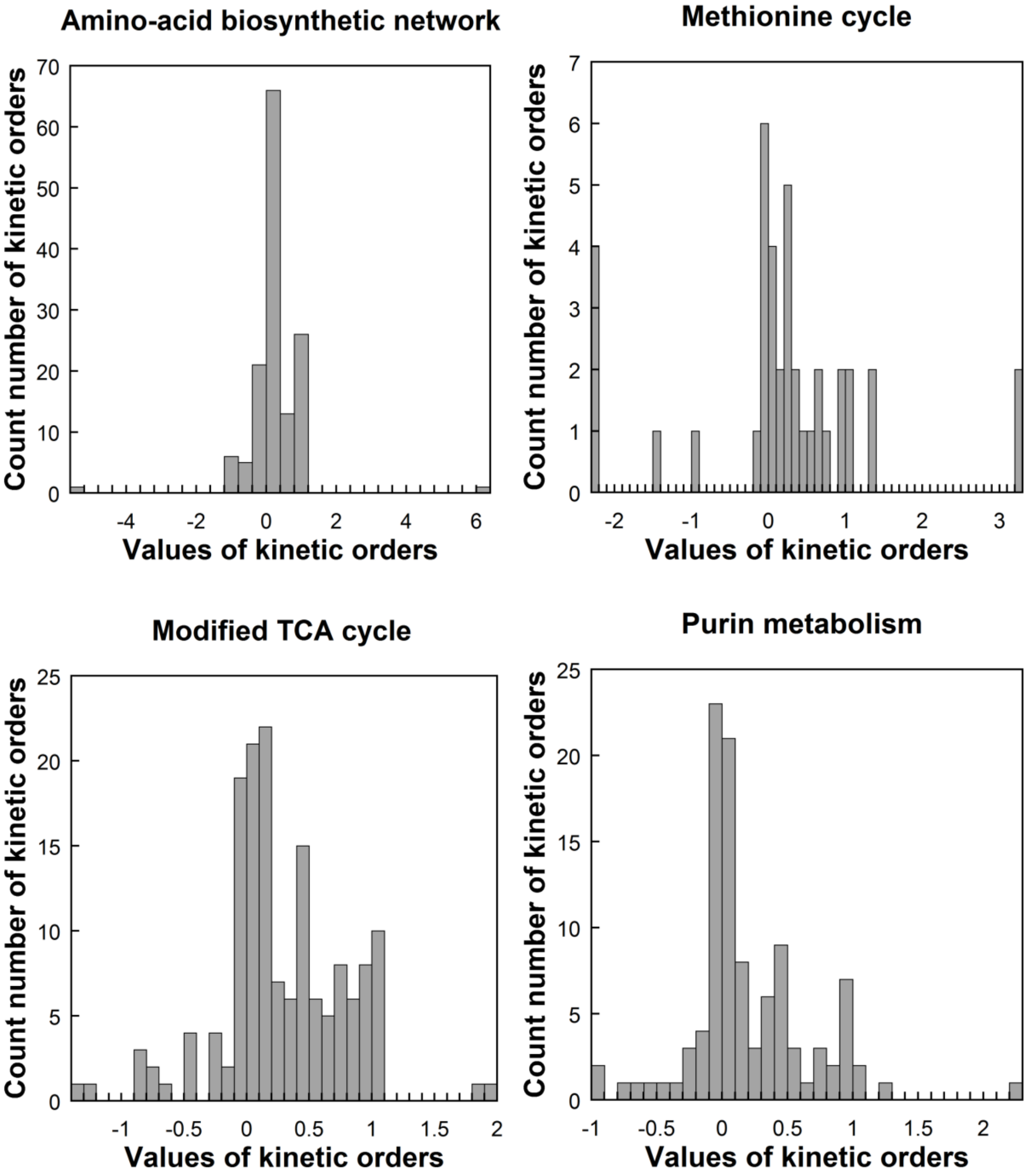

Fig. S3 Count numbers of values of kinetic orders for several models: (1) Amino acid biosynthetic network (Stephanopoulos and Simpson, 1997), (2) Methionine cycle (Reed, et al., 2004), (3) Modified TCA cycle (Shiraishi and Savageau, 1992), (4) Purin metabolism (Curto, et al., 1998)

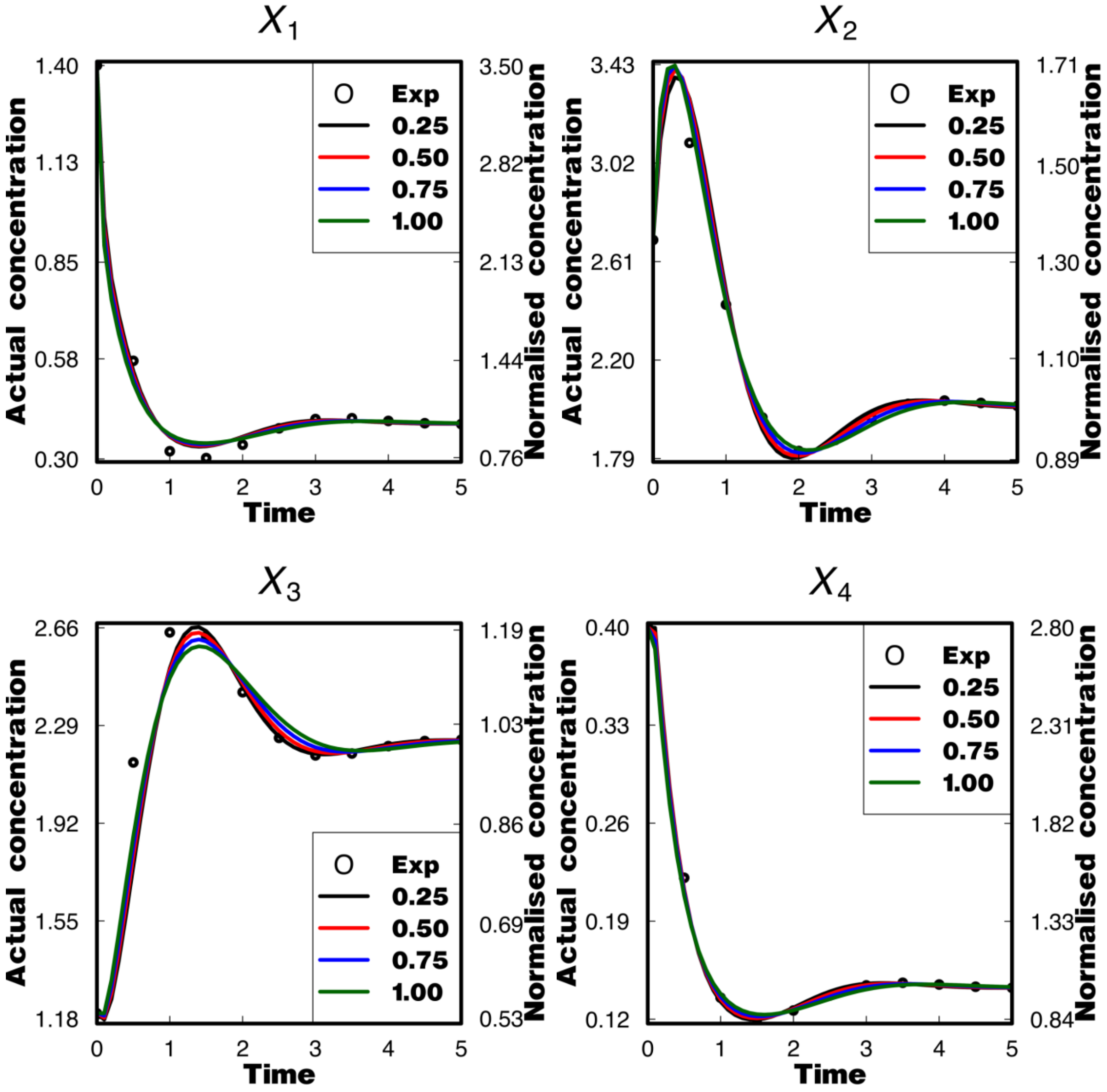

Fig. S4 Comparisons of the calculated lines based on 2 estimated parameters for kinetic orders of 0.25 or –0.25 (black lines), kinetic orders of 0.50 or –0.50 (red lines), kinetic orders of 0.75 or –0.75 (blue lines), kinetic orders of 1.0 or –1.0 (green lines) with 11 time-series data for each metabolite in a branched pathway model with inhibition and activation

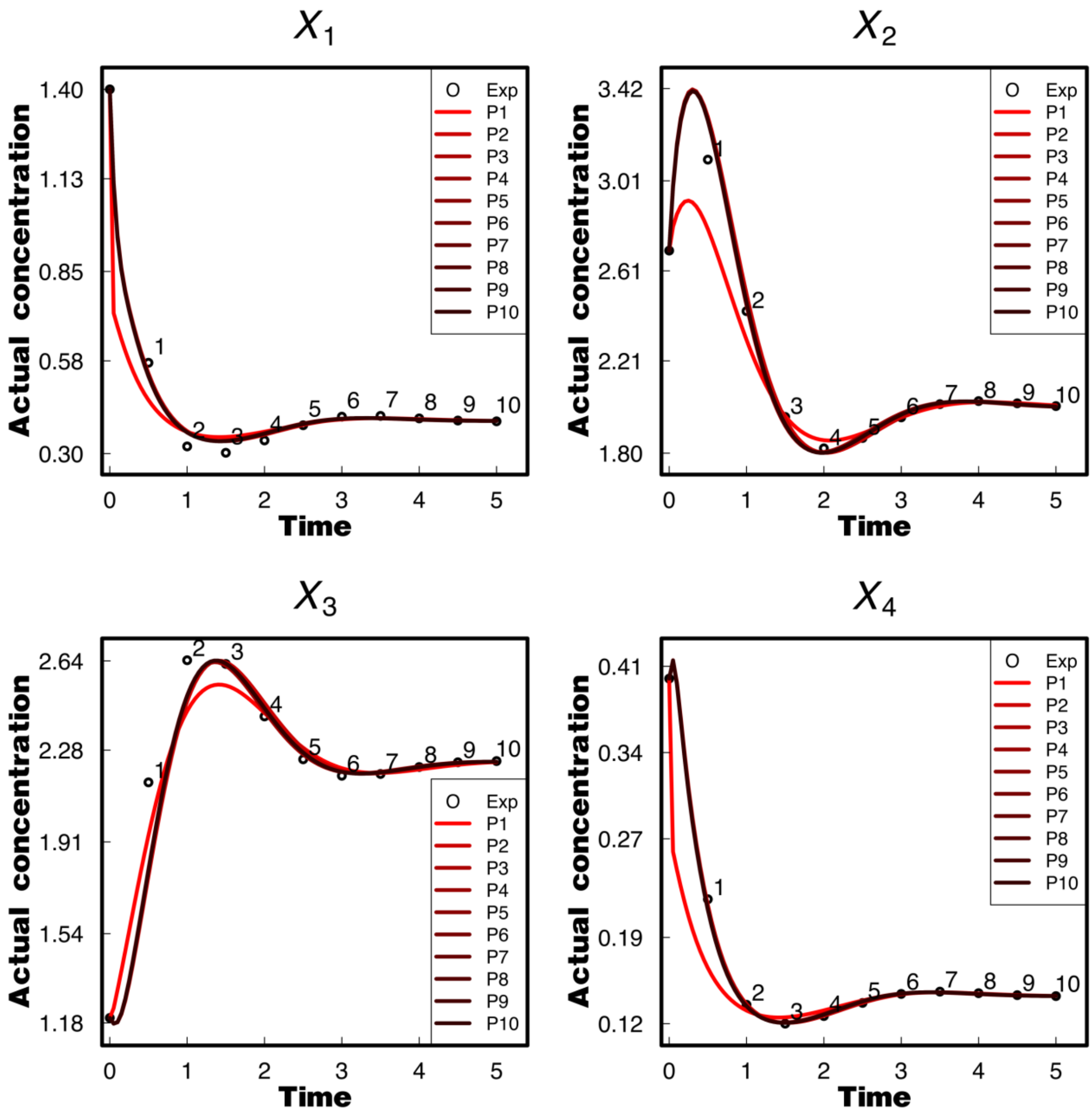

Fig. S5 Comparisons of the calculated lines based on 2 estimated parameters while one point of the time-series data taken from 11 time-series data for all metabolites were removed one-by-one (from data point 1 to 10 with calculated lines P1 to P10, respectively) in a branched pathway model with inhibition and activation

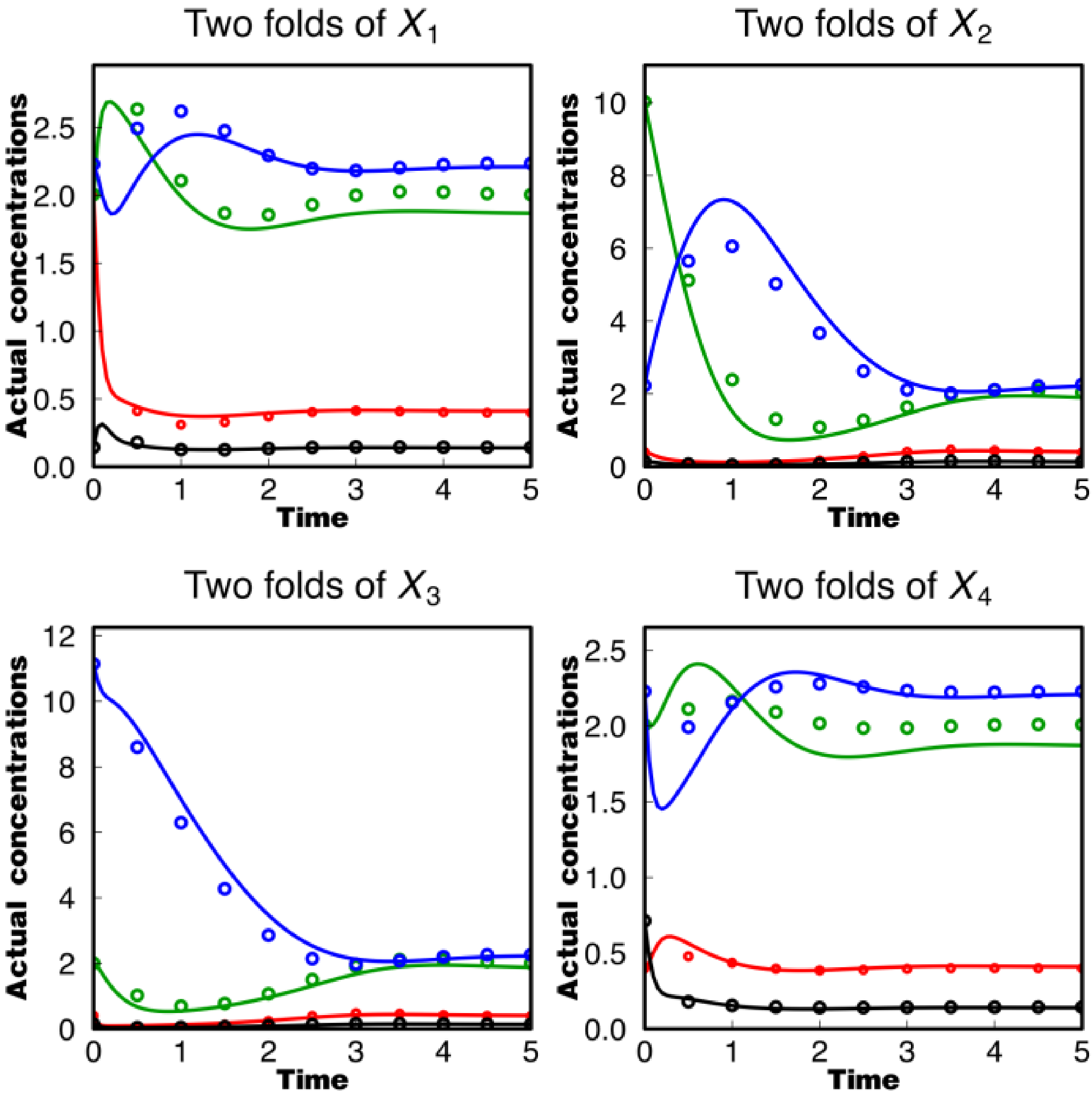

Fig. S6 Predictions of metabolic behaviors after perturbing X_1 (red), X_2 (green), X_3 (blue) and X_4 (black), respectively, two-folds at t=0 using the model constructed by PENDISC (simulation lines) compared with *in silico* 11 time-series data generated by the model with actual parameter values for both rate constants and kinetic orders

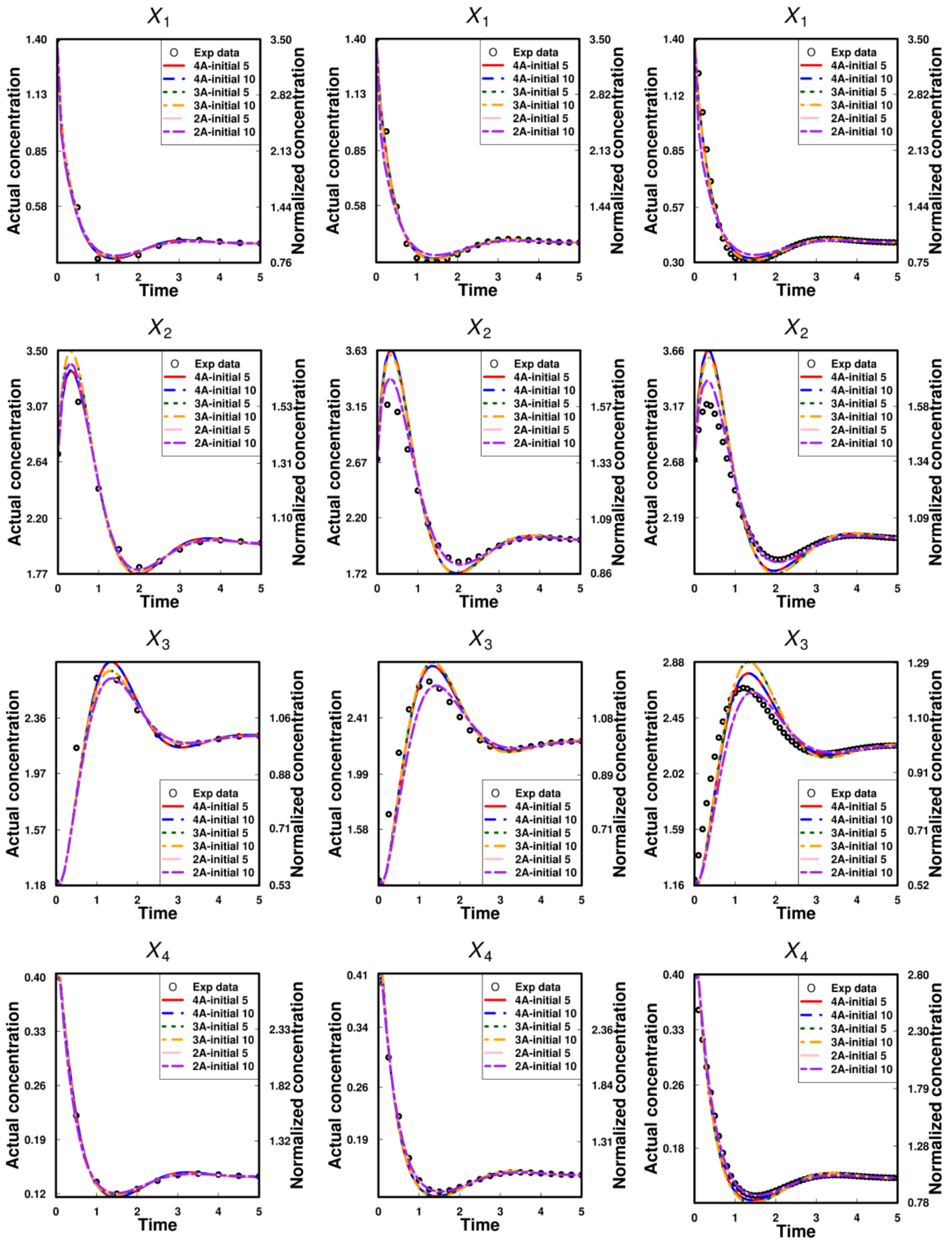

Fig. S7 Comparisons of calculated lines based on two, three, and four estimated parameters (represented by the symbols “2A,” “3A,” and “4A,” respectively) with 11, 21 and 51 time-series data for each metabolite in a branched metabolic pathway model with inhibition and activation. The left, middle, and right columns represent the results for different numbers of time-series data. The initial guesses were all set to either 5 or 10 (represented by the symbols “initial 5” and “initial 10,” respectively).

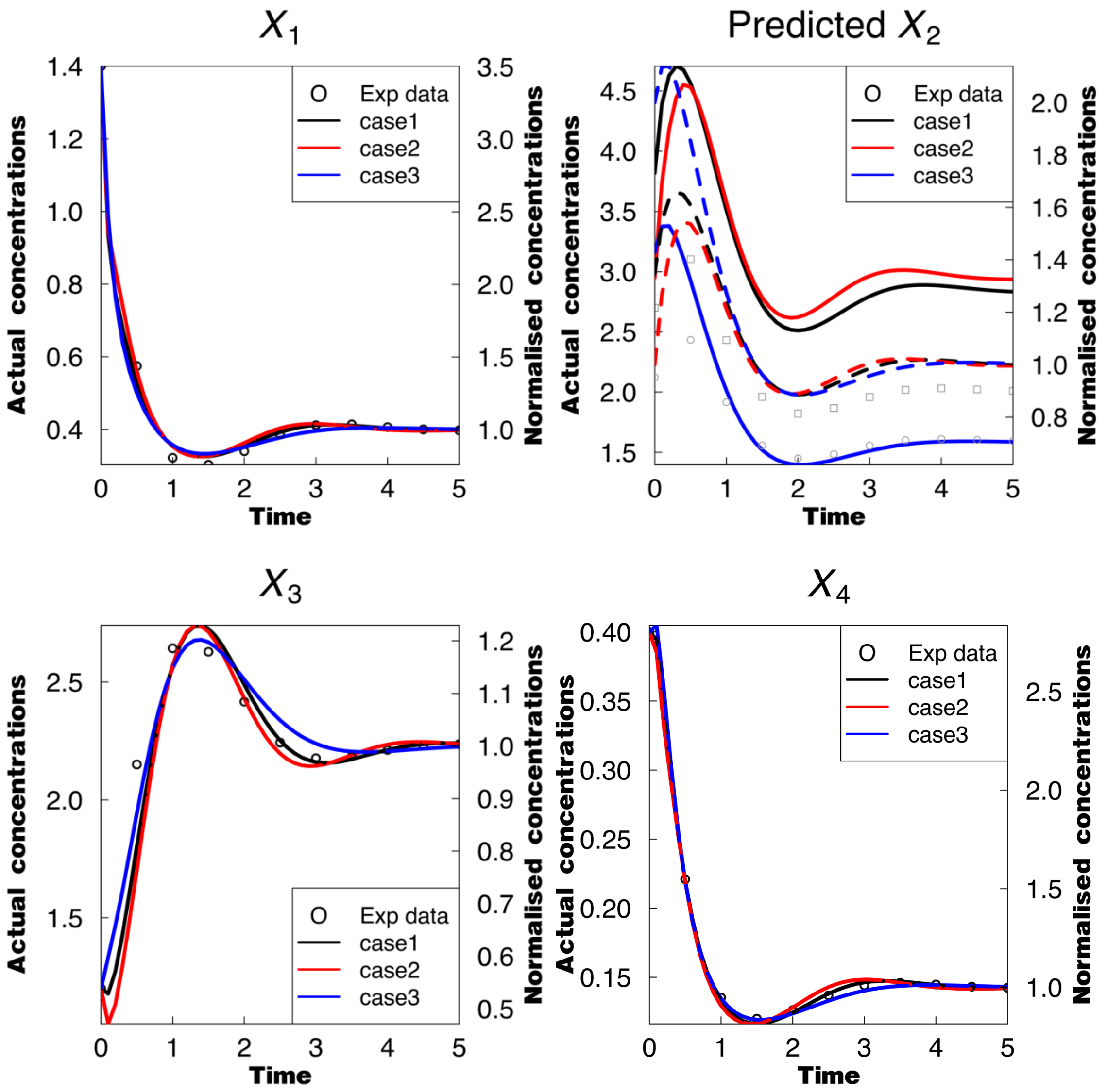

Fig. S8 Comparisons of calculated lines based on estimated 2 parameters with 11 time-series data for each metabolite except X_2 in a branched pathway model with inhibition and activation using different initial values of X_2 (x_{20} for case 1=2.7/2.006073, x_{20} for case 2=1.0 and x_{20} for case 3=2.0, respectively). Solid lines indicate actual concentrations whereas broken lines indicate normalized concentrations.

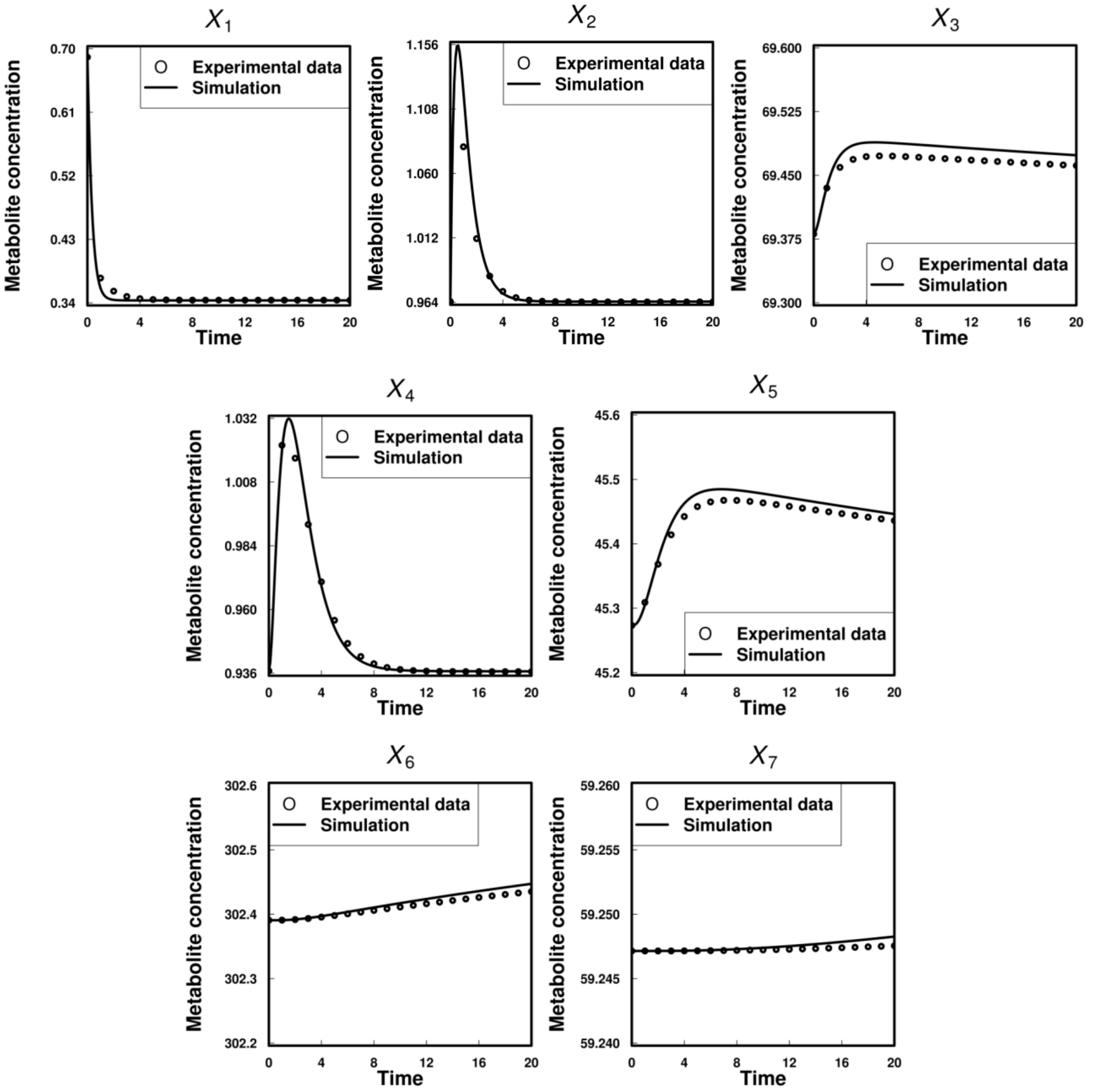

Fig. S9 Comparisons of calculated lines based on 2 estimated parameters with 21 time-series data for each metabolite in an aspartate-derived amino acid biosynthesis model. The initial guesses were all set to be 5.

References

Curien, G., *et al.* (2009) Understanding the regulation of aspartate metabolism using a model based on measured kinetic parameters, *Mol Syst Biol*, **5**.

Curto, R., *et al.* (1998) Mathematical models of purine metabolism in man, *Math. Biosci.*, **151**, 1-49.

Reed, M.C., *et al.* (2004) A mathematical model of the methionine cycle, *J. Theor. Biol.*, **226**, 33-43.

Shiraishi, F. and Savageau, M. (1992) The tricarboxylic acid cycle in Dictyostelium discoideum. I.Formulation of alternative kinetic representations, *J Biol Chem*, **267**, 22912-22918.

Stephanopoulos, G.N. and Simpson, T.W. (1997) Flux amplification in complex metabolic networks, *Chem. Eng. Sci*, **52**, 2607-2627.

Voit, E.O. and Almeida, J. (2004) Decoupling dynamical systems for pathway identification from metabolic profiles, *Bioinformatics*, **20**, 1670-1681.
